# Acetylcarnitine shuttling links mitochondrial metabolism to histone acetylation and lipogenesis

**DOI:** 10.1126/sciadv.adf0115

**Published:** 2023-05-03

**Authors:** Luke T. Izzo, Sophie Trefely, Christina Demetriadou, Jack M. Drummond, Takuya Mizukami, Nina Kuprasertkul, Aimee T. Farria, Phuong T. T. Nguyen, Nivitha Murali, Lauren Reich, Daniel S. Kantner, Joshua Shaffer, Hayley Affronti, Alessandro Carrer, Andrew Andrews, Brian C. Capell, Nathaniel W. Snyder, Kathryn E. Wellen

**Affiliations:** ^1^ Department of Cancer Biology, University of Pennsylvania, Philadelphia, PA 19104, USA.; ^2^ Abramson Family Cancer Research Institute, University of Pennsylvania, Philadelphia, PA 19104, USA.; ^3^ Center for Metabolic Disease Research, Lewis Katz School of Medicine, Temple University, Philadelphia, PA 19140, USA.; ^4^ Department of Cancer Epigenetics, Fox Chase Cancer Center, Philadelphia, PA 19111, USA.; ^5^ Department of Dermatology, Perelman School of Medicine, University of Pennsylvania, Philadelphia, PA 19104, USA.; ^6^ Department of Chemistry and Biochemistry, University of North Carolina Wilmington, Wilmington, NC 28403, USA.

## Abstract

The metabolite acetyl-CoA is necessary for both lipid synthesis in the cytosol and histone acetylation in the nucleus. The two canonical precursors to acetyl-CoA in the nuclear-cytoplasmic compartment are citrate and acetate, which are processed to acetyl-CoA by ATP-citrate lyase (ACLY) and acyl-CoA synthetase short-chain 2 (ACSS2), respectively. It is unclear whether other substantial routes to nuclear-cytosolic acetyl-CoA exist. To investigate this, we generated cancer cell lines lacking both ACLY and ACSS2 [double knockout (DKO) cells]. Using stable isotope tracing, we show that both glucose and fatty acids contribute to acetyl-CoA pools and histone acetylation in DKO cells and that acetylcarnitine shuttling can transfer two-carbon units from mitochondria to cytosol. Further, in the absence of ACLY, glucose can feed fatty acid synthesis in a carnitine responsive and carnitine acetyltransferase (CrAT)-dependent manner. The data define acetylcarnitine as an ACLY- and ACSS2-independent precursor to nuclear-cytosolic acetyl-CoA that can support acetylation, fatty acid synthesis, and cell growth.

## Teaser

Acetylcarnitine shuttling from mitochondria can supply acetyl-CoA for lipogenesis and acetylation independent of ACLY and ACSS2.

## INTRODUCTION

Acetyl-CoA is a central metabolic intermediate that is generated during nutrient catabolism, used as a building block for lipid synthesis, and serves as the substrate for protein and metabolite acetylation. In mitochondria, acetyl-CoA is generated from the breakdown of nutrients including carbohydrates, fatty acids, and amino acids. Mitochondrial acetyl-CoA enters the tricarboxylic acid cycle through a condensation reaction with oxaloacetate to generate citrate. The mitochondrial pool of acetyl-CoA is spatially distinct from acetyl-CoA found in the nucleus and cytosol due to the inability of acetyl-CoA to cross the inner mitochondrial membrane. Because of this compartmentalization, acetyl-CoA must be generated separately within the nucleus or cytosol for its use in these compartments. This is accomplished by ATP-citrate lyase (ACLY), which cleaves citrate exported from mitochondria into acetyl-CoA and oxaloacetate, and acyl-CoA short chain fatty acid synthase 2 (ACSS2), which condenses acetate with coenzyme A. Acetyl-CoA generated by these enzymes is used for de novo lipogenesis (DNL), as well as for acetylation in the nucleus and cytosol (*1*–*3*). Metabolites including acetyl-CoA can diffuse through nuclear pores but can also be generated within the nucleus (*1*).

**Fig. 1.**
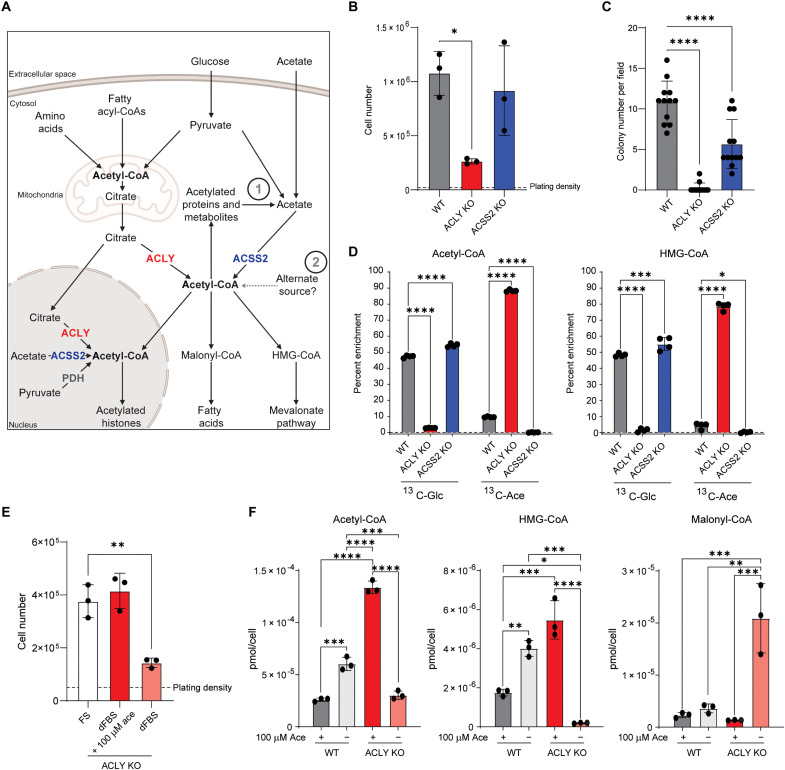
ACLY KO cancer cells proliferate in the absence of exogenous acetate. (**A**) Schematic diagram of acetyl-CoA metabolism. Arrows represent biochemical conversions. Numbers refer to potential ACLY- and exogenous acetate-independent acetyl-CoA generating pathways in the nuclear-cytosolic compartment. Created with BioRender.com. (**B**) Proliferation of WT, ACLY KO, and ACSS2 KO HCC cell lines over 4 days in Dulbecco’s modified Eagle’s medium (DMEM) + 10% FS. Statistical significance was calculated by one-way analysis of variance (ANOVA). (**C**) Colony formation in soft agar of WT, ACLY KO, and ACSS2 KO HCC cells. Colonies were counted at ×4 magnification. Data are from three replicate wells with four fields counted per well. Statistical significance was calculated by one-way ANOVA. (**D**) Whole cell acetyl-CoA and HMG-CoA percent carbon enrichment in WT, ACLY KO, and ACSS2 KO HCC cells cultured in glucose- and glutamine-free DMEM + 10% dFBS + either 10 mM ^13^C_6_-glucose (^13^C-Glc) + 100 μM acetate or 10 mM glucose + 100 μM ^13^C_2_-acetate (^13^C-Ace) for 6 hours. Statistical significance was calculated by two-way ANOVA. (**E**) ACLY KO proliferation in DMEM + 10% FS, DMEM + 10% dFBS, or DMEM + 10% dFBS ± 100 μM acetate for 96 hours. Statistical significance was calculated by one-way ANOVA. (**F**) Whole-cell acyl-CoA measurements of WT and ACLY KO cells grown in DMEM + 10% dFBS or DMEM + 10% dFBS ± 100 μM acetate for 24 hours. Statistical significance was calculated by two-way ANOVA comparing all conditions and genotypes. FS, full serum (10% calf serum); dFBS, dialyzed FBS. Each point represents a biological replicate, and error bars represent SD. *P ≤ 0.05; **P ≤ 0.01; ***P ≤ 0.001; ****P ≤ 0.0001.

We previously demonstrated that in the absence of ACLY, ACSS2 is up-regulated and acetate feeds acetyl-CoA pools for lipogenesis and histone acetylation (*4*). Furthermore, *Acly^−/−^* mouse embryonic fibroblasts (MEFs) are dependent on exogenous acetate for viability, suggesting that acetyl-CoA synthesis by ACSS2 is the primary compensatory mechanism in the absence of ACLY (*4*). Such compensation from acetate via ACSS2 is also observed in vivo upon deletion of *Acly* from adipose tissue or liver (*4*–*6*). Yet, several clues suggested that additional acetyl-CoA generating mechanisms within the nuclear-cytosolic compartment must exist. First, in the absence of ACLY, about 20 to 40% of the acetyl-CoA pool in whole cells and in the cytosol remains unlabeled from exogenous acetate (*4*, *7*). Second, histone acetylation is suppressed in the absence of ACLY at physiological acetate concentrations but does not appear to decline further when acetate is withdrawn (*4*). Last, although glucose use for fatty acid synthesis is strongly suppressed in the absence of ACLY, it is not fully blocked in vitro or in vivo (*4*, *6*). On the basis of these clues, we hypothesized that two broad mechanisms could potentially account for these findings: (i) intracellular acetate production from other nutrients and/or (ii) an acetyl-CoA producer other than ACLY and ACSS2 (Fig. 1A).

Both types of mechanisms have been reported, but the significance of such pathways remains poorly understood. In terms of endogenous sources, acetate production can occur directly from pyruvate non-enzymatically or via the pyruvate dehydrogenase complex (PDC) (*8*, *9*). In addition, acetate can be released from histone deacetylation (*10*) and acetylated metabolite hydrolysis (*11*–*13*). Furthermore, noncanonical routes to acetyl-CoA production outside of mitochondria have also been proposed, with several studies reporting a moonlighting function of the PDC, which can translocate to the nucleus under specific conditions to generate a local source of acetyl-CoA from pyruvate for histone acetylation (*14*–*17*). Beyond pyruvate to acetate conversion and nuclear PDC, less well understood routes of nuclear-cytosolic acetyl-CoA metabolism have been suggested, including peroxisomal production and export of acetyl-CoA and acetylcarnitine shuttling out of the mitochondria (*18*–*20*). The functional significance of such pathways relative to the canonical pathways via ACLY and ACSS2 remains poorly understood.

To evaluate potential alternative acetyl-CoA–producing pathways, we generated cancer cell lines in which *Acly* and *Acss2* are genetically deleted individually or in combination. Intriguingly, we show that in contrast to fibroblasts (*4*), cancer cells lacking both ACLY and ACSS2 [double knockout (DKO) cells] are viable, proliferate, contain a nuclear-cytosolic pool of acetyl-CoA, and sustain protein acetylation. Using carbon-13 tracing experiments, we demonstrate that fatty acids and glucose are prominent sources of acetyl-CoA that can feed acetyl-CoA pools and histone acetylation in the absence of ACLY and ACSS2. The data indicate that for glucose, this is mediated at least in part via the carnitine shuttle and carnitine acetyltransferase (CrAT). In the absence of ACLY, acetyl-units can be transported out of the mitochondria as acetylcarnitine to enable cytosolic acetyl-CoA synthesis and DNL from glucose-derived carbon. Acetylcarnitine also promotes histone acetylation and supports cell proliferation in a manner dependent on the lysine acetyltransferase (KAT) p300 in cells lacking ACLY and ACSS2. While CrAT is necessary for the generation of acetylcarnitine from acetyl-CoA in mitochondria, conversion of mitochondrially derived or exogenous acetylcarnitine back to acetyl-CoA for histone acetylation and cytosolic DNL is CrAT-independent. Overall, the data demonstrate that ACLY and ACSS2 are not the sole sources of acetyl-CoA in the nuclear-cytosolic compartment and that acetylcarnitine shuttling can participate in the transit of acetyl-CoA from mitochondria to cytosol to support DNL, histone acetylation, and proliferation.

## RESULTS

### Cancer cells maintain viability and proliferation in the absence of ACLY and exogenous acetate

Since cancer cells are adept at engaging available metabolic flexibility mechanisms, we hypothesized that noncanonical acetyl-CoA production mechanisms could be revealed by developing ACLY KO cancer cell models that are not dependent on acetate for viability. We used murine *Acly^flox/flox^* hepatocellular carcinoma (HCC) cell lines and generated isogenic cell lines lacking ACLY or ACSS2 via adenoviral Cre treatment or CRISPR-Cas9 gene editing, respectively (fig. S1A). ACLY KO cells had a markedly decreased proliferation rate in cell growth and soft agar colony formation assays (Fig. 1, B and C). ACSS2 KO cells had no impairment of two-dimensional (2D) cell growth but showed a modest defect in the numbers and size of soft agar colonies formed compared to parental controls (Fig. 1, B and C, and fig. S1B). As expected, loss of ACLY potently suppressed uniformly labeled ^13^C_6_-glucose (^13^C-Glc) and increased ^13^C_2_-acetate (^13^C-Ace) incorporation into acetyl-CoA and the downstream metabolite 3-hydroxy-3-methylglutaryl (HMG)-CoA (Fig. 1D). ACSS2 KO cells used glucose similarly to their wild-type (WT) counterparts, and labeling from acetate was completely ablated, showing that ACSS2 is the primary acyl-CoA synthetase that mediates synthesis of acetyl-CoA from acetate in these cells (Fig. 1D). These data functionally validate our KO cell models.

We next tested whether ACLY loss rendered these cancer cells reliant on acetate for proliferation and acetyl-CoA synthesis. While proliferation slows in ACLY KO HCC cells in the absence of acetate, the cells retain viability and continue to proliferate, in contrast to ACLY KO MEFs (Fig. 1E and fig. S1C) (*4*). Similar observations were made in another cancer cell line, derived from Kras^G12D^-driven pancreatic cancer in mice with *Acly* deletion in the pancreas (*21*), which also could proliferate in the absence of ACLY and exogenous acetate (fig. S1D). These ACLY KO pancreatic cancer cells showed little to no reliance on exogenous acetate for proliferation, which is similar to that previously observed in ACLY null glioblastoma cells (*4*). In the HCC ACLY KO cells, we quantified acetyl-CoA in the presence or absence of acetate, finding that acetyl-CoA abundance is elevated in ACLY KO cells in the presence of acetate and decreases back to that in WT cells in its absence (Fig. 1F). In contrast, HMG-CoA is almost entirely depleted upon acetate withdrawal, consistent with that reported previously in ACLY KO cells (*22*). Unexpectedly, malonyl-CoA is elevated under these conditions (Fig. 1F). Together, these findings indicate that while exogenous acetate feeds acetyl-CoA pools in the absence of ACLY, additional mechanisms must also be available to cells to support proliferation.

### Cancer cells proliferate and maintain a pool of cytosolic acetyl-CoA in the absence of both ACLY and ACSS2

**Fig. 2.**
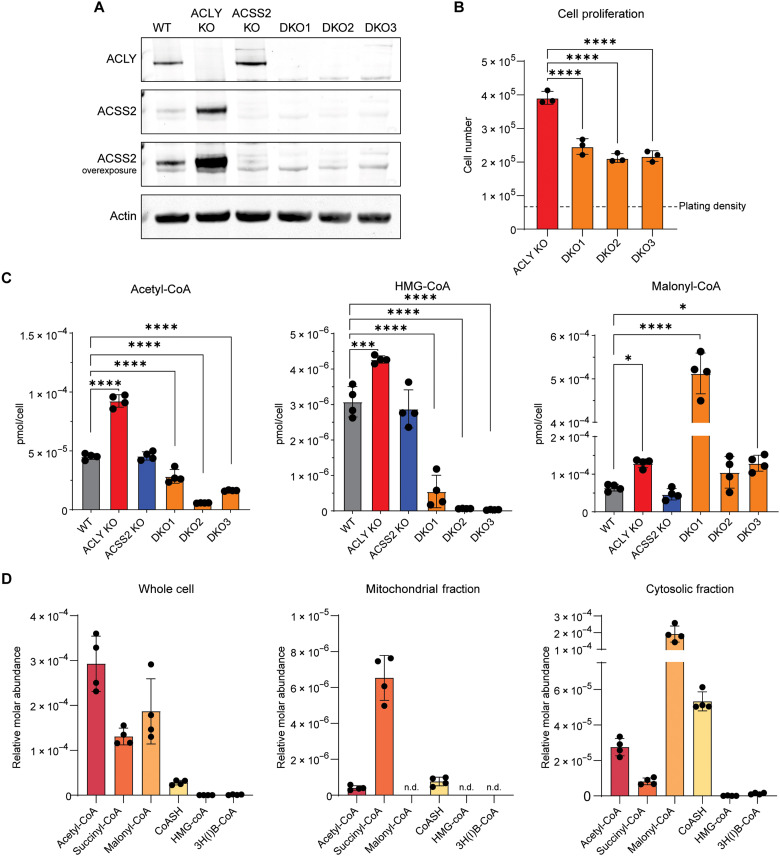
ACLY/ACSS2 DKO cancer cells are viable and maintain a cytosolic pool of acetyl-CoA. (**A**) Western blot for ACLY and ACSS2 in WT, ACLY KO, ACSS2 KO, and three DKO cell lines. (**B**) Proliferation of ACLY KO and DKO HCC cell lines after 5 days in DMEM + 10% FS. Data are represented as mean of three replicates ± SD. Statistical significance was calculated by one-way ANOVA between samples. (**C**) Whole-cell acyl-CoA measurements in WT, ACLY KO, ACSS2 KO, and DKO cell lines grown in DMEM + 10% dFBS + 100 μM acetate for 24 hours. Statistical significance was calculated by one-way ANOVA. (**D**) Acyl-CoA quantitation using SILEC-SF performed on DKO1 cells grown in DMEM + 10% dFBS + 100 μM acetate for 24 hours. 3H(I)B-CoA, 3-HB-CoA, and (iso)butyryl-CoA are not resolved and are represented together. Acyl-CoA species marked with n.d. were not detected in *n* = 4 samples. Abundance of acyl-CoAs within a given compartment can be compared to one another. Each point represents a biological replicate, and error bars represent SD. *P ≤ 0.05; ***P ≤ 0.001; ****P ≤ 0.0001.

One possible explanation for these data is an endogenous source of acetate. To test this, we asked whether ACSS2 is required for viability in ACLY KO cells, using CRISPR-Cas9 gene editing to delete *Acss2* in the ACLY KO HCC and pancreatic ACLY KO cells. Single-cell clonal selection revealed that cells lacking both ACLY and ACSS2 (DKO) are viable and proliferate, albeit slower (Fig. 2, A and B, and fig. S2, A and B). Even in conditions that are known to trigger ACSS2 up-regulation [hypoxia and low serum (*23*)], no residual ACSS2 protein was detected in the DKO cells (fig. S2C).

We next examined acyl-CoA abundance, finding that acetyl-CoA is modestly reduced, HMG-CoA is markedly reduced, and malonyl-CoA tends to be elevated in DKO cells, similar to the phenotype seen in ACLY KO cells in the absence of acetate (Fig. 2C and fig. S2D). To determine whether a substantial acetyl-CoA pool is present in the cytosol of DKO cells, we applied stable isotope labeling of essential nutrients in cell culture–subcellular fractionation (SILEC-SF), a recently developed technique for rigorous quantification of acyl-CoAs in subcellular compartments that allows for comparison between acyl-CoA species within a subcellular compartment (*22*). Subcellular measurements demonstrated distinct acyl-CoA profiles in mitochondria versus cytosol, including a clear cytosolic acetyl-CoA pool in the DKO cells and comparable compartment-specific acetyl-CoA levels between DKO and ACLY KO (Fig. 2D and fig. S2E). The data indicate that cells are able to maintain nuclear-cytosolic acetyl-CoA pools independent of ACLY and ACSS2.

### Cells lacking ACLY and ACSS2 are dependent on exogenous fatty acids

**Fig. 3.**
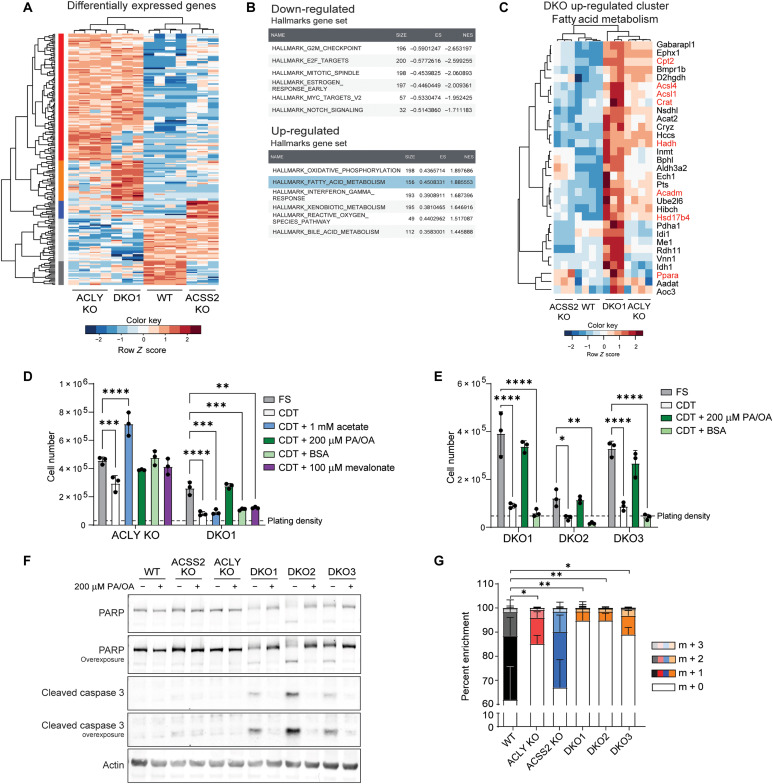
ACLY and ACSS2 deficiency alters fatty acid metabolism and causes reliance on exogenous fatty acids. (**A**) Heatmap of all differentially expressed genes between all four genotypes, log_2_ fold change > 2 and an adjusted *P* value < 0.01 expressed as row *Z*-score. DESeq counts were log_2_-transformed before clustering. Row clusters (red, orange, blue, light gray, and dark gray) represent groups of genes commonly differentially regulated by sample cluster. See table S1 for gene list by cluster. (**B**) The top six up-regulated and down-regulated Hallmarks gene sets from GSEA analysis comparing DKO cells to all other genotypes. ES, enrichment score; NES, normalized enrichment score. (**C**) Genes from the hallmarks fatty acid metabolism gene set commonly up-regulated across DKO1 samples. Cluster expanded from fig. S3D. DESeq counts were log_2_-transformed before clustering. (**D** and **E**) Cell proliferation after 96 hours. Cells were plated in DMEM/F12 media overnight and then cultured in DMEM + 10% FS or CDT serum with or without the addition of metabolites. PA/OA is 100 μM of each fatty acid conjugated to bovine serum albumin (BSA; 200 μM total). BSA condition is equal volume of fatty acid–free BSA as added to PA/OA condition. Statistical significance was calculated by two-way ANOVA. (**F**) Western blot analysis of cells cultured in DMEM + 10% CDT serum with or without PA/OA. PA/OA is 100 μM of each fatty acid conjugated to BSA (200 μM total). Without PA/OA conditions contain fatty acid–free BSA. PARP, poly(adenosine diphosphate–ribose) polymerase. (**G**) Isotopologue enrichment of palmitate measured by GC-MS. Cells were cultured in DMEM + 10% D_2_O + 10% dFBS +100 μM acetate for 24 hours. Bars represent mean of three or four biological replicates for each cell line. Statistical analysis performed on total hydrogen enrichment. Statistical significance was calculated by one-way ANOVA. CDT, charcoal dextran treated. Each point represents a biological replicate, and error bars represent SD. *P ≤ 0.05; **P ≤ 0.01; ***P ≤ 0.001; ****P ≤ 0.0001.

Having established the existence of an extramitochondrial acetyl-CoA pool in the DKO cells, we carried out RNA sequencing (RNA-seq) to identify potential compensatory pathways. Principal components analysis revealed distinct separation between ACLY-deficient (ACLY KO and DKO1) and ACLY-proficient genotypes (WT and ACSS2 KO) (fig. S3A). Both ACLY KO and DKO cells showed marked transcriptional changes compared to WT with substantial overlap. However, there was also a distinct subset of genes specifically regulated in the DKO cells. (Fig. 3A; fig. S3, B and C; and table S1). Thus, we performed gene set enrichment analysis (GSEA) to identify functional groups of genes up- or down-regulated in the DKO cells (Fig. 3B). Cell cycle–related genes were among those suppressed, while fatty acid metabolism was notably enriched in the DKO cells, including fatty acid oxidation–related genes (Fig. 3, B and C, and fig. S3D). These included both mitochondrial and peroxisomal fatty acid oxidation genes (Fig. 3C). These data suggest that lipid metabolism may be perturbed in the DKO cells and that fatty acid oxidation pathways might be up-regulated as part of a compensatory mechanism.

To begin to investigate functional changes in lipid metabolism, we first tested whether loss of ACLY and ACSS2 resulted in dependence on exogenous lipids. For this, DKO cells were cultured in media supplemented with serum treated with charcoal dextran (CDT), a process that removes lipophilic compounds. DKO cells cultured in these lipid-depleted conditions failed to proliferate and began to undergo apoptosis (Fig. 3, D to F), while their ACLY KO counterparts proliferated with only a slight defect (Fig. 3D). DKO cells also failed to proliferate in dialyzed fetal bovine serum (dFBS), which removes small molecules using a 10,000–molecular weight cutoff membrane (fig. S3G). Acetate increased proliferation in ACLY KO cells but had no effect on DKO cells (Fig. 3D and fig. S3E). Addition of bovine serum albumin (BSA)–conjugated palmitic and oleic acids (PA/OA) fully rescued proliferation of DKO cells, and either PA or OA alone was also sufficient (Fig. 3, D and E, and fig. S3, E to G). Supplementation of mevalonate, mevalonate-phosphate, or the medium chain fatty acid octanoate failed to rescue DKO cell proliferation (Fig. 3D and fig. S3E). Thus, cells lacking ACLY and ACSS2 are dependent on exogenous fatty acids for viability and proliferation.

We next asked whether this requirement for exogenous fatty acids reflected a limited ability to synthesize fatty acids de novo. Since the pathways and carbon sources supplying acetyl-CoA in the DKO cells were unknown, DKO cells were cultured in the presence of deuterated water, and deuterium incorporation into palmitate was used to examine total DNL. Compared to WT and ACSS2 KO cells, DKO cells exhibited very low palmitate labeling (Fig. 3G and fig. S3H). This limited DNL suggests that DKO cells might rely on exogenous fatty acids due to reduced ability to adequately synthesize their own, despite maintaining higher or similar concentrations of malonyl-CoA (Fig. 2C).

### Fatty acids regulate histone acetylation in the absence of ACLY and ACSS2

**Fig. 4.**
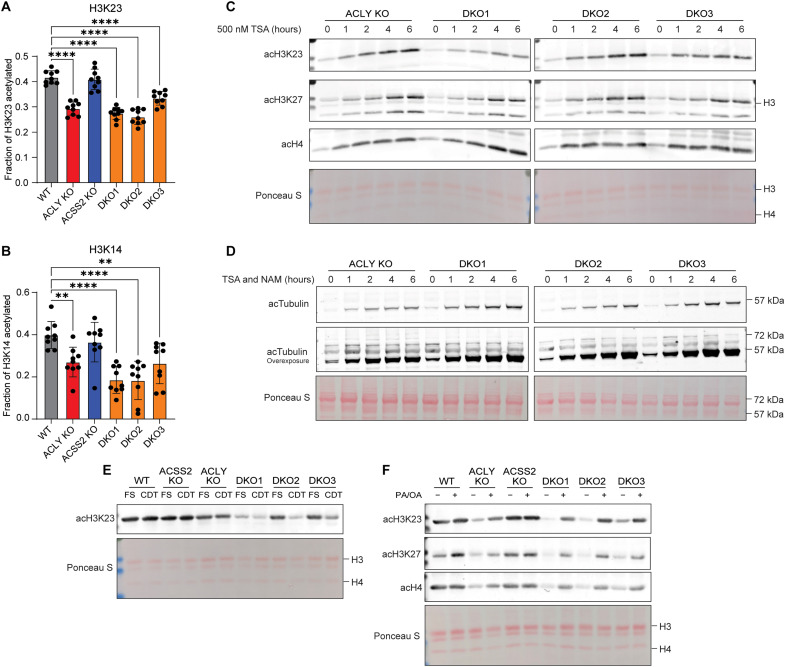
Fatty acid availability modulates histone acetylation independent of ACLY and ACSS2. (**A**) Fraction of the total quantified H3K23 residues acetylated by LC-MS. Statistical significance was calculated by one-way ANOVA. (**B**) Fraction of the total quantified H3K14 residues acetylated by LC-MS. Statistical significance was calculated by one-way ANOVA. (**C**) Acid-extracted histone Western blot from cells grown in DMEM + 10% FS and treated with 500 nM TSA over a time course. Ponceau S stain for total protein in histone extracts used for Western blot. (**D**) Whole-cell protein extract Western blot from cells grown in DMEM + 10% FS and treated with 500 nM TSA and 500 μM nicotinamide (NAM) over a time course. Ponceau S stain for total protein in histone extracts used for Western blot. (**E**) Acid-extracted histone Western blot from cells cultured in DMEM + 10% FS or CDT for 24 hours. Ponceau S stain for total protein in histone extracts used for Western blot. (**F**) Acid-extracted histone Western blot from cells cultured for 24 hours in DMEM + 10% CDT and supplemented with PA/OA. PA/OA is 100 μM of each fatty acid conjugated to BSA (200 μM total). Ponceau S stain for total protein in histone extracts used for Western blot. Each point represents a biological replicate, and error bars represent SD. **P ≤ 0.01; ****P ≤ 0.0001.

To further understand how acetyl-CoA is used in the DKO cells, we assessed levels of histone acetylation, another major nutrient-sensitive acetyl-CoA–dependent process (*1*), across the four genotypes. We quantified acetylation at sites on histone H3 by mass spectrometry, focusing on two high abundance acetylation sites that have been proposed as acetate reservoirs, H3K23ac and H3K14ac (*24*). Consistent with prior studies (*4*, *25*), ACLY KO cells maintain lower levels of histone acetylation at H3K23 and H3K14 than their WT and ACSS2 KO counterparts, and DKO cells exhibit similar levels of acetylation as single ACLY KO cells (Fig. 4, A and B). To investigate whether the capacity of DKO cells to acetylate is intact, we blocked histone deacetylation with the broad HDAC inhibitor trichostatin A (TSA). Here, we analyzed the putative reservoir site H3K23ac, the regulatory site H3K27ac, and pan acetyl-H4. TSA treatment causes an increase in global histone acetylation in all marks analyzed over time in ACLY KO cells, and this effect is comparable in DKO cells (Fig. 4C). This suggests that acetyl-CoA is readily available for use for histone acetylation in cells lacking both ACLY and ACSS2. Similarly, tubulin acetylation and total lysine acetylation dynamics, as assessed by a pan K-ac antibody, were comparable between genotypes (Fig. 4D and fig. S4A) upon treatment with TSA and the sirtuin inhibitor nicotinamide (NAM), indicating that acetyl-CoA is available in both the nucleus and cytosol in these cells.

We hypothesized that serum lipids might play a role in sustaining histone acetylation independent of ACLY and ACSS2, because fatty acid oxidation produces acetyl-CoA and genes involved in oxidation are up-regulated in DKO cells. To test this, DKO cells were incubated in lipid-depleted culture conditions. Lipid depletion led to a marked depletion of histone acetylation within 24 hours, and this was rescued by addition of PA/OA (Fig. 4, E and F, and fig. S4B). Acetyl-CoA abundance increased modestly with PA/OA supplementation, with some variability between lines (fig. S4C). Malonyl-CoA, on the other hand, was potently suppressed by fatty acid supplementation (fig. S4D). This is consistent with the known role of fatty acyl-CoAs in allosteric inhibition of the acetyl-CoA–consuming enzyme ACC (*26*, *27*), which may divert acetyl-CoA toward histone acetylation, as previously reported (*28*–*30*). Consistently, ACC inhibition (ND630) also modestly increased histone acetylation in the DKO cells while suppressing malonyl-CoA levels (fig. S4, D and E), indicating that fatty acids may act to increase histone acetylation in part through acetyl-CoA sparing from ACC consumption. We also tested octanoate, which has been previously shown to promote histone acetylation (*31*), and found that octanoate also increased histone acetylation and acetyl-CoA abundance in the DKO cells, although did not rescue proliferation (figs. S3E and S4, B, F, and G). Together, these data show that exogenous fatty acids can regulate histone acetylation levels independently of ACLY and ACSS2.

### Fatty acids and glucose can feed acetyl-CoA pools and histone acetylation independent of ACLY and ACSS2

**Fig. 5.**
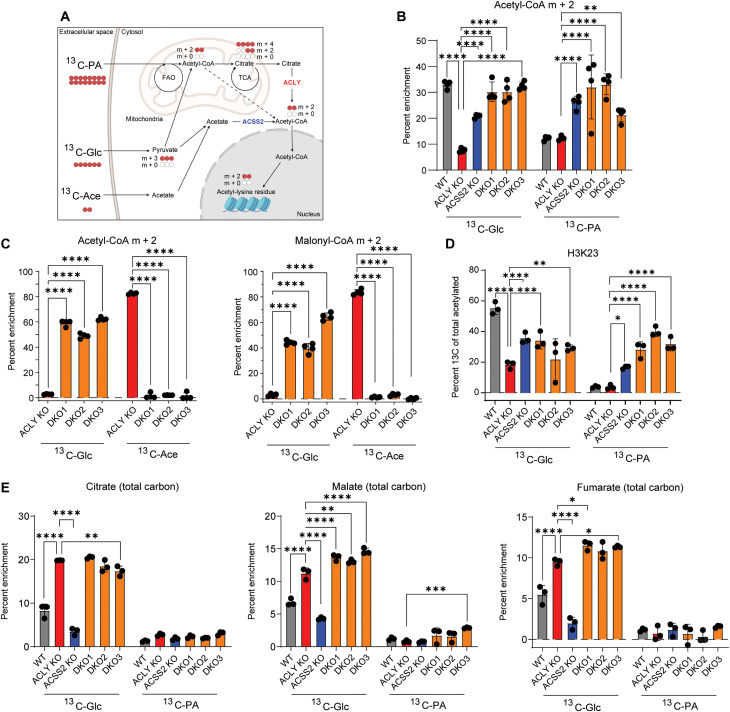
Fatty acids and glucose can supply acetyl-CoA for histone acetylation in a manner independent of ACLY and ACSS2. (**A**) Schematic depicting glucose, palmitate, and acetate carbon tracing into acetyl-CoA and histone acetylation. Created with BioRender.com. (**B**) ^13^C-Glc and ^13^C_16_-palmitate tracing into acetyl-CoA analyzed by LC-MS. Cells were cultured in glucose- and glutamine-free DMEM + 10% CDT supplemented with 4 mM glutamine and either 10 mM ^13^C-Glc and 100 μM palmitate conjugated to BSA or 10 mM glucose and 100 μM ^13^C_16_-palmitate conjugated to BSA for 2 hours. Statistical significance was calculated by two-way ANOVA. (**C**) Acetyl-CoA and malonyl-CoA enrichment from ^13^C-Glc or ^13^C-Ace. Cells were cultured in glucose- and glutamine-free DMEM + 10% dFBS supplemented with 4 mM glutamine and either 10 mM ^13^C-Glc and 100 μM acetate or 10 mM glucose and 100 μM ^13^C-Ace for 6 hours. Statistical significance was calculated by two-way ANOVA. (**D**) ^13^C-Glc and ^13^C_16_-palmitate tracing into acetylation on extracted histones, analyzed by LC-MS. Cells were cultured in glucose- and glutamine-free DMEM + 10% CDT supplemented with 4 mM glutamine and either 10 mM ^13^C-Glc and 100 μM palmitate conjugated to BSA or 10 mM glucose and 100 μM ^13^C_16_-palmitate conjugated to BSA for 24 hours. Statistical significance was calculated by two-way ANOVA. (**E**) ^13^C-Glc and ^13^C_16_-palmitate tracing into TCA cycle intermediates, analyzed by GC-MS. Cells were cultured in glucose- and glutamine-free DMEM + 10% CDT supplemented with 4 mM glutamine and either 10 mM ^13^C-Glc and 100 μM palmitate conjugated to BSA or 10 mM glucose and 100 μM ^13^C_16_-palmitate conjugated to BSA for 6 hours. Statistical significance was calculated by two-way ANOVA. Each point represents a biological replicate, and error bars represent SD. All tests compared to ACLY KO as the control. *P ≤ 0.05; **P ≤ 0.01; ***P ≤ 0.001; ****P ≤ 0.0001.

To determine whether fatty acid oxidation contributes substantially to the acetyl-CoA pool in DKO cells, we used stable isotope tracing of uniformly labeled ^13^C palmitate (^13^C_16_-PA) into whole-cell acetyl-CoA pools (Fig. 5A). A time-course experiment showed maximal labeling of acetyl-CoA in DKO cells within 2 hours, reaching up to 30% (fig. S5A). These findings suggest rapid and substantial breakdown of palmitate into acetyl-CoA. We also examined labeling from ^13^C-Glc, unexpectedly finding that although glucose was a minor contributor to acetyl-CoA pools in ACLY KO cells, it labeled a much greater fraction, similar to that of palmitate, in DKO cells (Fig. 5B). Under these conditions, whole-cell acetyl-CoA abundance was similar between genotypes (fig. S5B). In addition, in the absence of fatty acids, glucose labeled nearly 60% of the acetyl-CoA pool in DKO cells, although the pool size was reduced (Fig. 5C and fig. S5C). Malonyl-CoA accumulates in the absence of fatty acids in these cells in an ACC-dependent manner (fig. S4D) and labeling paralleled that of acetyl-CoA, suggesting that glucose-derived carbon may feed into an extramitochondrial acetyl-CoA pool in these cells (Fig. 5C). Acetate did not detectably contribute to acetyl-CoA pools in DKO cells (Fig. 5C). Thus, both glucose and fatty acids are major carbon sources feeding acetyl-CoA pools in the absence of ACLY and ACSS2.

To determine whether acetyl-CoA generated from glucose and palmitate is used for acetylation in the nucleus, we performed liquid chromatography–mass spectrometry (LC-MS) analysis of histone acetylation in cells incubated with isotope-labeled glucose or palmitate. WT cells used glucose-derived carbons for histone acetylation, and this was blunted by ACLY KO as expected (Fig. 5D). ACSS2 KO slightly blunted glucose carbon incorporation into histone acetylation, possibly reflecting the recycling of acetyl-groups over the 24-hour time (*10*). DKO cells had greater histone acetylation labeling from glucose than ACLY KO, in line with acetyl-CoA labeling. In addition, palmitate-derived carbon was used prominently by ACSS2 KO and DKO cells. The cumulative fractional labeling from palmitate and glucose in DKO cell histone acetylation matches that by WT and ACSS2 KO cells, while ACLY KO cells have much lower cumulative labeling from these two sources, consistent with unlabeled acetate feeding the acetyl-CoA pool only in those cells (fig. S5D). Together, these data indicate that both glucose and fatty acids can supply nuclear-cytosolic acetyl-CoA through a pathway that does not require ACLY or ACSS2 and that can be used for histone acetylation.

### ACLY KO cells have altered glucose usage in the TCA cycle

We next sought to define the mechanism(s) of ACLY- and ACSS2-independent production of nuclear-cytosolic acetyl-CoA. Since both glucose and fatty acids could contribute to histone acetylation, we reasoned that this could occur either by two distinct substrate-specific mechanisms or a mechanism in which these substrates can feed into a common precursor acetyl-CoA pool (e.g., in mitochondria) that is then transported to the nuclear-cytosolic compartment (fig. S5E).

Given multiple publications documenting a nuclear PDC (*14*–*17*), we investigated the localization of PDH in the HCC cells. Using immunofluorescence and confocal microscopy, we observed prominent mitochondrial but minimal nuclear localization of catalytic subunit of the PDC (PDHe1α) (fig. S6A). In addition, PDHe1α protein levels are unchanged in standard culture conditions and are unaffected by fatty acid availability (fig. S6, B and C). While not formally ruling out a role for a nuclear PDC in this context, the data suggest that a different mechanism likely sustains the DKO cells.

We next asked whether a mitochondrial route might be involved since both glucose and fatty acids can feed mitochondrial acetyl-CoA pools (Fig. 5A and fig. S5E). ^13^C-glucose labeling into tricarboxylic acid (TCA) cycle intermediates was elevated in ACLY KO and DKO cells despite modest to no change in abundance of these metabolites (Fig. 5E and fig. S5F). Palmitate was poorly used as a carbon source in the TCA cycle in all genotypes, suggesting that fatty acid oxidation in the mitochondria is a more minor source of mitochondrial acetyl-CoA (Fig. 5E). Inhibition of mitochondrial (etomoxir) and peroxisomal (thioridazine) fatty acid oxidation revealed that each organelle contributes to approximately half of acetyl-CoA produced from palmitate in the DKO cells (fig. S5G). Notably, inhibition of fatty acid oxidation is insufficient to prevent fatty acid induced histone acetylation, suggesting that fatty acids regulate histone acetylation partially through mechanisms independent of supplying acetyl-CoA, such as inhibition of ACC, as noted above (fig. S5H). Together, the data indicate that glucose-derived carbon feeds the mitochondrial metabolite pool and suggest the mitochondria as a possible intermediate location for glucose carbons before supplying nuclear-cytosolic acetyl-CoA in DKO cells.

### Acetylcarnitine shuttling facilitates glucose-dependent lipogenesis in the absence of ACLY

Acetyl-CoA cannot directly cross organelle membranes, and thus, a transport mechanism out of mitochondria and/or peroxisomes is needed to explain the data. We noticed that *Crat* mRNA, encoding the CrAT, is increased in DKO cells (Fig. 3C). CrAT is present in mitochondria and peroxisomes (*32*, *33*) and transfers the acetyl moiety from acetyl-CoA onto carnitine to generate acetylcarnitine for organelle export via the carnitine-acylcarnitine translocase (CACT; SLC25A20). CrAT is thought to play a buffering function to prevent high levels of mitochondrial acetyl-CoA, which can suppress pyruvate oxidation and cause non-enzymatic acetylation (*34*–*36*). Some evidence suggests that acetylcarnitine can be converted back to acetyl-CoA within the cytosol and nucleus, although such a pathway remains poorly understood (*19*, *37*–*39*).

**Fig. 6.**
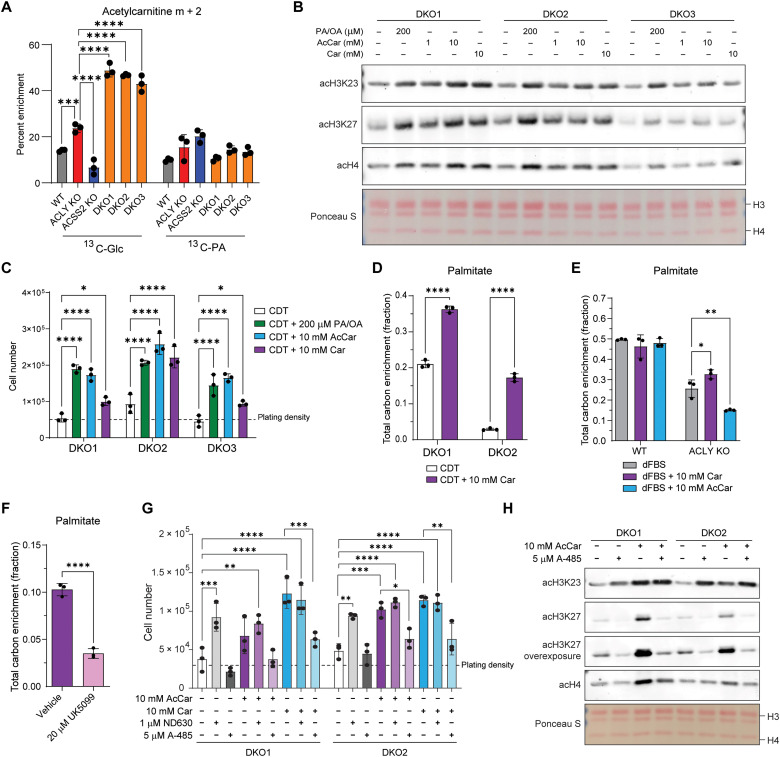
Carnitine facilitates histone acetylation and DNL from glucose-derived carbon. (**A**) ^13^C-Glc and ^13^C_16_-palmitate tracing into acetylcarnitine, analyzed by LC-MS. Cells were cultured in glucose- and glutamine-free DMEM + 10% CDT with 4 mM glutamine and either 10 mM ^13^C-Glc and 100 μM palmitate or 10 mM glucose and 100 μM ^13^C_16_-palmitate for 6 hours. Statistical significance was calculated by two-way ANOVA. (**B**) Acid-extracted histone Western blot from cells cultured in DMEM + 10% CDT for 24 hours supplemented with PA/OA, acetylcarnitine, or carnitine. PA/OA is 100 μM of each fatty acid (200 μM total). Ponceau S stain for total protein in histone extracts used for Western blot. (**C**) Cell proliferation after 96 hours. Cells were plated in DMEM/F12 media overnight then cultured in DMEM + 10% CDT serum with or without the addition of metabolites. PA/OA is 100 μM of each fatty acid (200 μM total). Statistical significance was calculated by two-way ANOVA. (**D** to **F**) ^13^C-Glc tracing into palmitate measured by GC-MS. Cells were cultured in glucose- and glutamine-free DMEM + 10% CDT or dFBS supplemented with 4 mM glutamine and 10 mM ^13^C-Glc with or without 10 mM carnitine (D and E) or with 10 mM carnitine with vehicle control or 20 μM UK5099 (F) for 48 hours. Statistical significance was calculated by unpaired *t* tests (D and F) or two-way ANOVA (E). (**G**) Cell proliferation after 96 hours as in (C) or without the addition of metabolites or inhibitors. Statistical significance was calculated by two-way ANOVA comparing all conditions within genotypes, and not all significant comparisons are shown. (**H**) Acid-extracted histone Western blot as in (B) supplemented with acetylcarnitine and/or p300 inhibition. Each point represents a biological replicate, and error bars represent SD. *P ≤ 0.05; **P ≤ 0.01; ***P ≤ 0.001; ****P ≤ 0.0001.

To determine whether acetylcarnitine could serve as a metabolic intermediate in the generation of acetyl-CoA in the nuclear-cytosolic compartment in DKO cells, we traced palmitate and glucose into acetylcarnitine. Acetylcarnitine is highly labeled from glucose in ACLY KO and DKO (Fig. 6A), similar to TCA cycle labeling, suggesting that acetylcarnitine reflects and may be in equilibrium with the mitochondrial acetyl-CoA pool. Palmitate labels the acetylcarnitine pool but to a lesser extent than glucose; thus, it may be that fatty acids—potentially via peroxisomal oxidation—can supply acetyl-CoA to the nuclear-cytosolic compartment independent of the carnitine shuttle (Fig. 6A). Nevertheless, these findings prompted us to ask whether CrAT provides a mechanism for shuttling of acetyl-units, especially those derived from glucose, into the nuclear-cytosolic acetyl-CoA pool.

If CrAT can generate acetyl-CoA for histone acetylation, then we anticipated that the supplementation of acetylcarnitine to the culture media should boost histone acetylation and proliferation in lipid-depleted DKO cells, and this was indeed the case (Fig. 6, B and C). Notably, supplementation of DKO cells with l-carnitine alone was also able to increase both histone acetylation and cell proliferation in lipid-depleted conditions (Fig. 6, B and C), suggesting that it may promote acetylcarnitine shuttling out of mitochondria.

To further probe this possibility, we tested whether l-carnitine supplementation increased the ability of DKO cells to carry out DNL. Using deuterated water labeling, we found that l-carnitine significantly increased synthesis of palmitate and stearate in DKO cells (fig. S7, A and B). In addition, supplementation of l-carnitine enhanced glucose-dependent DNL in the absence of ACLY and ACSS2, implicating the carnitine shuttle in transport of acetyl-units out of mitochondria (Fig. 6D and fig. S7C).

To understand whether carnitine can also promote glucose-dependent DNL outside of the DKO context, we tested the impact of carnitine supplementation on WT and ACLY KO cells. WT cell DNL from glucose was unaffected by carnitine supplementation (Fig. 6E and fig. S7, D to G). ACLY KO cells, however, showed a significant increase in glucose-derived DNL when supplemented with carnitine (Fig. 6E and fig. S7, D to G). In addition, supplementation of acetylcarnitine further reduced glucose-dependent DNL in ACLY KO cells, suggesting that the acetyl-units provided by acetylcarnitine supplementation dilute the glucose-derived acetyl-CoA pools (Fig. 6E and fig. S7, D to F). Carnitine and acetylcarnitine supplementation did not affect fatty acid abundance in any genotype (fig. S7, H and I).

Next, we tested whether the mitochondrial entry of pyruvate was necessary for carnitine driven glucose labeling of fatty acids in ACLY KO cells by inhibiting the mitochondrial pyruvate carrier (MPC). Inhibition of MPC by UK5099 causes a marked decrease in glucose contribution to DNL (Fig. 6F and fig. S7K). Notably, UK5099 in combination with carnitine supplementation appeared to cause toxicity (fig. S7L). In addition, glucose incorporation into whole-cell acetyl-CoA was increased in ACLY KO and DKO cells with carnitine supplementation, and acetylcarnitine suppressed glucose-dependent labeling, particularly in ACLY KO cells (fig. S7J). Together, these findings show that acetyl-units from the mitochondria can be exported and used in the cytosol for DNL independent of ACLY in a manner facilitated by carnitine.

We next asked which downstream fate(s) of acetyl-CoA are required to rescue the growth defects in lipid-depleted conditions in DKO cells. To address this, we treated DKO cells with either the ACC inhibitor ND630 to suppress malonyl-CoA formation for DNL (fig. S4D) or the p300/CBP inhibitor A-485. A-485 treatment potently suppressed acetylation of H3K27 but not H3K23 [which is mediated by KAT6A/B (*40*)] in response to acetylcarnitine supplementation (Fig. 6H). Treatment of lipid-depleted DKO cells with ND630 enhanced proliferation (Fig. 6G), coinciding with an increase in histone acetylation (fig. S4E). Further, ND630 did not suppress the l-carnitine- or acetylcarnitine-mediated increase in cell proliferation (Fig. 6G). On the other hand, treatment with A-485 blocked the proliferation rescue by l-carnitine and acetylcarnitine (Fig. 6G). Together, these data suggest that p300-dependent acetylation rather than DNL is the primary downstream fate of acetyl-CoA necessary for maintaining cell proliferation in this context.

### CrAT is essential for glucose-dependent ACLY-independent DNL

**Fig. 7.**
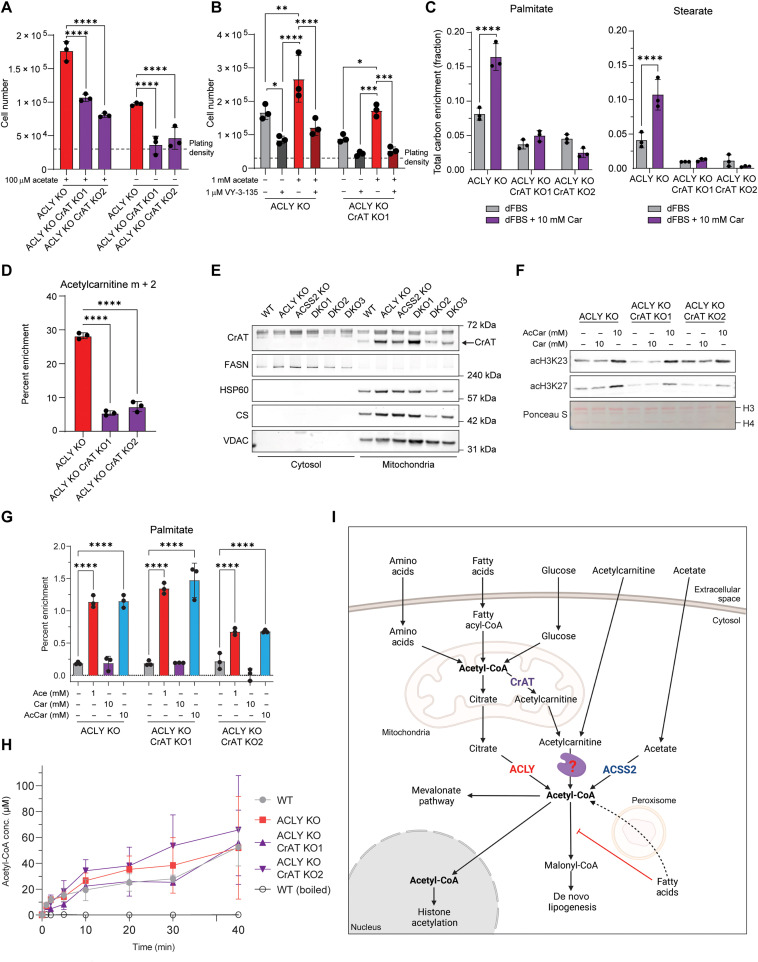
Acetylcarnitine shuttling provides acetyl-units to the nuclear cytosolic compartment. (**A** and **B**) Cell proliferation after 96 hours. Cells were plated in DMEM/F12 media overnight and then cultured in DMEM + 10% dFBS ± 100 μM acetate (A) or DMEM + 10% dFBS ± 1 mM acetate and or the ACSS2 inhibitor VY-3-135 (B). Statistical significance was calculated by two-way ANOVA (A) or two-way ANOVA comparing all conditions within genotypes (B). (**C**) ^13^C-Glc tracing into fatty acids measured by GC-MS. Cells were cultured in glucose- and glutamine-free DMEM + 10% dFBS supplemented with 4 mM glutamine and 10 mM ^13^C-Glc with or without 10 mM carnitine for 48 hours. Statistical significance was calculated by unpaired *t* tests. (**D**) ^13^C-Glc tracing into acetylcarnitine, analyzed by LC-MS. Cells were cultured in glucose- and glutamine-free DMEM + 10% dFBS supplemented with 4 mM glutamine and 10 mM ^13^C-Glc for 6 hours. Statistical significance was calculated by one-way ANOVA. (**E**) Mitochondrial fractionation of HCC cells and Western blotting for CrAT, a cytosolic marker (FASN), and mitochondrial markers (HSP60, CS, and VDAC). The band above CrAT is nonspecific. (**F**) Acid-extracted histone Western blot from cells cultured in DMEM + 10% dFBS supplemented with or without carnitine or acetylcarnitine for 24 hours. (**G**) Deuterium tracing into palmitate measured by GC-MS. Cells were cultured in DMEM + 10% D_2_O + 10% dFBS ± 10 mM acetate, carnitine, or acetylcarnitine for 24 hours. Statistical significance was calculated by two-way ANOVA. (**H**) In vitro CrAT activity assay. Production of acetyl-CoA from acetylcarnitine is shown over time. (**I**) Schematic depicting CrAT-dependent acetylcarnitine shuttling out of the mitochondria for acetyl-CoA generation in the nuclear-cytosolic compartment. Created with BioRender.com. Each point represents a biological replicate, and error bars represent SD. *P ≤ 0.05; **P ≤ 0.01; ***P ≤ 0.001; ****P ≤ 0.0001.

We next asked whether CrAT was necessary for glucose carbon contribution to acetyl-CoA–dependent processes in the nucleus and cytosol in the absence of ACLY. To address this, we generated clonal CrAT KO cells from either ACLY KO (fig. S8, A and B) or WT (parental) cells (fig. S8C). We noted that CrAT deficiency slowed proliferation in both WT and ACLY KO cells (Fig. 7A and fig. S8, D and E). The ability of ACLY KO cells to proliferate in acetate-deprived conditions was lost in the absence of CrAT, consistent with the CrAT-acetylcarnitine and acetate-ACSS2 pathways representing the two major pathways of acetyl-CoA generation that can support growth in the absence of ACLY (Fig. 7A). In addition, ACSS2 inhibition with VY-3-135 (*41*) blocked the ability of acetate to rescue proliferation in cells lacking ACLY and CrAT, while CrAT-proficient ACLY KO cells retain some ability to proliferate with ACSS2 inhibition (Fig. 7B).

To test whether CrAT is necessary for the carnitine-mediated shuttling of acetyl-units out of the mitochondria, glucose labeling into fatty acids was assessed. CrAT deficiency suppressed the residual glucose-dependent DNL in ACLY KO cells and abrogated the effect of carnitine supplementation in promoting DNL (Fig. 7C). Consistently, CrAT KO suppressed acetylcarnitine labeling from glucose (Fig. 7D). On the other hand, CrAT deficiency had no impact on DNL in ACLY-proficient cells, even when carnitine was supplemented, consistent with a dominant role for ACLY in two carbon transfer from mitochondria to cytosol (fig. S8F). Carnitine supplementation increased labeling of acetylcarnitine from fatty acids, but CrAT deficiency did not affect this, further suggesting that fatty acids engage a distinct route from glucose that is CrAT-independent (fig. S8G). Together, the data indicate that in the absence of ACLY, CrAT can mediate the transfer of mitochondrial acetyl-units to the nuclear-cytosolic space through facilitating acetyl-CoA to acetylcarnitine conversion.

One remaining question, however, is whether CrAT is present in the cytosol to mediate the conversion of acetylcarnitine back to acetyl-CoA in the cytosol. Therefore, we performed fractionation experiments to determine the subcellular localization of CrAT. Since CrAT is annotated as a mitochondrial protein, we fractionated cells to deplete the mitochondria from the cytosol to determine whether CrAT is present in the cytosolic compartment. CrAT is clearly present in the mitochondria but was not detectable in the cytosol (Fig. 7E and fig. S8H), suggesting that another enzyme might mediate acetyl-CoA regeneration in the cytosol. To further investigate this possibility, we treated CrAT KO cells with acetylcarnitine to determine whether CrAT was necessary for acetylcarnitine-driven histone acetylation. Acetylcarnitine boosts histone acetylation in ACLY KO cells in a CrAT-independent manner (Fig. 7F). Since ACLY KO CrAT KO cells express ACSS2, it is possible that the acetylcarnitine rescue in this setting reflects hydrolysis of acetylcarnitine to acetate. To control for this, cells were treated with an ACSS2 inhibitor, which revealed that while acetate depends on ACSS2 to promote histone acetylation, acetylcarnitine promotes histone acetylation in a manner independent of CrAT, ACSS2, and ACLY, although ACSS2 inhibition did slightly suppress the effect (fig. S8I). Acetylcarnitine also increases total DNL in ACLY KO CrAT KO cells, similar to acetate, suggesting that acetylcarnitine can generate lipogenic acetyl-CoA without CrAT (Fig. 7G and fig. S8J). Carnitine itself only increased histone acetylation and total DNL in DKO cells (Fig. 6B and fig. S7, A and B) and not in the ACLY single KO cells (Fig. 7G). Last, we performed in vitro CrAT activity assays in cell lysates derived from the HCC cells. Acetyl-CoA was generated from acetylcarnitine in cell lysates from WT, ACLY KO, and ACLY KO CrAT KO cells at similar rates, providing further evidence that CrAT is not required for the extramitochondrial generation of acetyl-CoA from acetylcarnitine (Fig. 7H). Boiled cell lysates were unable to generate acetyl-CoA from acetylcarnitine, indicating that this is likely an enzyme-catalyzed process. Together, these data indicate that CrAT is necessary for ACLY-independent transfer of two carbon units out of mitochondria but that it is likely not the enzyme that regenerates acetyl-CoA from acetylcarnitine in the nuclear-cytosolic compartment.

## DISCUSSION

The two well-established routes to nuclear-cytosolic acetyl-CoA pools used for lipid synthesis and histone acetylation are via mitochondrial citrate export and cleavage by ACLY and acetate activation by ACSS2. In this study, we demonstrate that the carnitine shuttle and CrAT can provide an alternative route for two carbon transport from mitochondria to cytosol to support histone acetylation, DNL, and proliferation (Fig. 7I). In cancer cell lines lacking both ACLY and ACSS2, we show that a pool of cytosolic acetyl-CoA is maintained, that histone acetylation is active, and that both glucose and fatty acids can supply acetyl-CoA. We further show that fatty acids boost histone acetylation both by serving as a carbon source and via acetyl-CoA sparing by ACC inhibition. Notably, our tracing data also suggest that a peroxisomal route is likely important for at least a portion of fatty acid–dependent acetyl-CoA production. This is confirmed and studied in depth in a complementary study reported in a manuscript co-submitted with this one (*42*). In the absence of ACLY, we show that carnitine supplementation increases DNL from glucose in a CrAT-dependent manner, thus demonstrating that the CrAT-acetylcarnitine pathway can be used as an ACLY-independent means of transporting two carbon units to the cytosol for use in acetyl-CoA–dependent processes, supporting cell proliferation in a manner dependent on p300/CBP (Fig. 7I). In addition, we show that CrAT is not necessary for regenerating acetyl-CoA from acetylcarnitine outside of mitochondria, which is consistent with a recent study that reported that carnitine octanoyltransferase (CrOT) and not CrAT produces acetyl-CoA from exogenous acetylcarnitine (*39*). Overall, this work broadens the understanding of nuclear-cytosolic acetyl-CoA metabolism and opens new avenues for investigation into the regulation of DNL and histone acetylation.

One reason for undertaking this study to identify compensatory mechanisms of acetyl-CoA metabolism is that both ACLY and ACSS2 are of interest as therapeutic targets. The liver-specific ACLY inhibitor bempedoic acid is U.S. Food and Drug Administration approved for LDL cholesterol lowering (*3*, *43*), and the first ACSS2 inhibitor has entered oncology clinical trials (NCT04990739). At least in mice, ACLY deletion (*6*) or bempedoic acid (*44*) strongly suppresses glucose-dependent hepatic DNL, suggesting that ACLY is a dominant mechanism for glucose-dependent DNL in the liver, although acetate can sustain rates of lipogenesis in the absence of ACLY (*6*). However, looking to the future, ACLY is also of interest as a potential target in oncology, and given the metabolic plasticity of cancer cells and the ability to shift between nutrient sources (*45*), potential roles of CrAT and CrOT should be considered. Carnitine is not only synthesized in the liver but also can be obtained through the diet. Carnitine is abundant in certain diets, such as those high in red meat, and the impact of dietary carnitine warrants exploration. In addition, l-carnitine and acetyl-l-carnitine dietary supplements are widely available. Inhibiting carnitine metabolism would need to be evaluated with caution, as carnitine deficiency can cause severe phenotypes including muscle wasting, heart failure, liver damage, and cognitive delays (*46*). In addition, it will be of interest to understand whether the carnitine shuttle or acetylcarnitine uptake plays a role in compensating for ACLY and ACSS2 under conditions such as high fat diet, in which both enzymes are suppressed in adipose tissue (*47*); presumably, acetyl-CoA would still be needed for histone acetylation and the mevalonate pathway even if fatty acid synthesis activity is low.

CrAT has mainly been studied for its role in mitochondrial metabolism, including fatty acid oxidation and acetyl-CoA buffering (*34*–*36*, *48*); however, there have been prior reports of CrAT outside of the mitochondria, as well as observations consistent with extramitochondrial CrAT activity. Early biochemical characterization of CrAT activity showed that it was at least partially localized on the cytosolic face of the endoplasmic reticulum membrane (*37*). Yet, most reports suggest that the CrAT enzyme is not a membrane-bound protein and is localized within the mitochondrial lumen and the peroxisome, predominantly due to different CrAT isoform expression (*33*). Tracing studies using labeled acetylcarnitine show that the acetyl-units from acetylcarnitine can indeed be used for lipid synthesis and that their usage is increased with ACLY inhibition by hydroxycitrate (*38*). Another study suggested that an isoform of CrAT is present in the nucleus and may promote histone acetylation in a manner dependent on the CACT (*19*). Last, acetyl-proteomics data in CrAT KO skeletal muscle showed that this perturbation increases mitochondrial acetylation, supporting the idea that CrAT can act as a buffer system for mitochondria acetyl-CoA but also observed decreases in cytosolic protein acetylation (*34*). In addition to acetyl-CoA, CrAT may also be important for shuttling of other short-chain acyl-CoAs from mitochondria to the nuclear-cytosolic compartment. This is consistent with evidence that odd chain and branched chain fatty acid synthesis is CrAT dependent (*49*) and that isoleucine catabolism, a mitochondrial process, can supply propionyl-CoA for histone propionylation and that this correlates with production of propionyl-carnitine (*22*). Nevertheless, despite these reports in the literature, the significance of such a route has not been widely appreciated, and the identity of the enzyme catalyzing the acetylcarnitine to acetyl-CoA reaction is not well documented. Our findings, using genetic models and isotope tracing, are consistent with a model in which CrAT produces acetylcarnitine from acetyl-CoA in mitochondria and then following export to the cytosol, a separate enzyme, potentially CrOT (*39*), converts acetylcarnitine back to acetyl-CoA as an alternative to ACLY for two carbon transfer from mitochondria (Fig. 7I). CrOT is a peroxisomal enzyme and more work is necessary to understand the interplay between peroxisomes and the cytosol in acetyl-CoA and acetylcarnitine metabolism.

Further work will be needed to characterize the physiological contexts in which this pathway is used versus the ACLY- or ACSS2-dependent routes to supply acetyl-CoA for lipid synthesis and chromatin modification. ACLY has been shown to participate in a noncanonical TCA cycle in a manner that influences cell fate (*50*). This depends on oxaloacetate production by the ACLY reaction, which is returned to the TCA cycle after conversion to malate by malate dehydrogenase. The carnitine shuttle offers a means to transport two carbon units to the cytosol without concomitant oxaloacetate production, and thus, one prediction is that this route might be important for supporting histone acetylation or lipogenesis in cell types that do not engage the noncanonical cycle. In addition, the ability of cells to generate nuclear-cytosolic acetyl-CoA from environmental acetylcarnitine without going through the mitochondria and ACLY might provide a way for cells to scavenge acetyl-units from the environment or for cell-to-cell cross-talk. Acetylcarnitine is present both in circulation and in tumor interstitial fluid, albeit at low micromolar levels (*51*).

An additional question emerging from this work is the function of histone acetate reservoirs. It has been proposed that histones provide a large reservoir of acetyl-units that can be mobilized either under metabolic stress or for a ready source of acetyl-CoA for site-specific histone acetylation and gene regulation (*24*, *52*, *53*). Lipid deprivation in the DKO cells causes a rapid depletion in global histone acetylation in DKO cells. This model could uniquely enable studies of the consequences of histone acetylation reservoir depletion for gene regulation and chromatin structure. In addition, DKO growth in lipid-depleted conditions appears to be regulated dominantly by p300-dependent acetylation. Understanding how histone acetylation in nutrient-starved contexts can control cell proliferation may have implications for cancer growth and therapy.

Overall, this work provides evidence that acetylcarnitine shuttling can contribute to lipid synthesis and histone acetylation to support cell proliferation. These data lay the groundwork for functional studies into the role of this pathway in physiological and disease contexts.

## MATERIALS AND METHODS

### Cell lines

Murine Acly^f/f^ HCC cell lines were generated from diethylnitrosamine-induced tumors in Acly^f/f^ mice and have been previously described (*22*). ACLY KO cells were generated by administration of adenoviral Cre recombinase, and single-cell clonal populations were generated by limiting serial dilution. WT and ACLY KO cells were then transduced with a LentiCRISPR v2 vector with no guide RNA or containing a guide RNA targeting the first exon of ACSS2 or near the active site of CrAT: ACSS2 KO, DKO1, DKO2 (and pancreatic cancer cell lines): mACSS2sg2, CGAGCTGCACCGGCGTTCTG; DKO3: mACSS2sg6, CTGCACCGGCGTTCTGTGG; CrAT KO1, CrAT KO2, CrAT KO3, ACLY KO CrAT KO1, and ACLY KO CrAT KO2: mCRATsg1, CACCGTCCACAAGTGCAACTATGGG.

Following transduction, cells were treated with puromycin until the entirety of an untransduced cell population died. Following puromycin treatment, single-cell clonal populations were generated for ACSS2 KO, CrAT KO, ACLY/ACSS2 DKO, and ACLY/CrAT DKO cells by limiting serial dilution. The bulk cell population of WT and ACLY KO cells transduced with the empty LentiCRISPR v2 was used as controls.

Murine pancreatic cancer cell lines were generated from pancreatic ductal adenocarcinoma tumors of Pdx1-Cre; LSL-Kras^G12D^; Tp53^f/f^; Acly^f/f^ mice (*21*). Mouse work was performed in accordance with Institutional Animal Care and Use Committee guidelines under an approved protocol. A female mouse with palpable tumors in the peritoneal cavity was euthanized at approximately 15 weeks of age. Pancreatic tumor was excised from the animal, minced in smaller pieces using sterile scissors, and lastly digested using a collagenase VI solution [2 mg/ml in Dulbecco’s modified Eagle’s medium (DMEM)/F12; Sigma-Aldrich, #C9891] for 20 min at 37°C. The solution was then filtered through a 70-μM mesh to obtain a single-cell suspension. Cells were cultured in pancreatic ductal epithelial cell (PDEC) medium (*21*) and tested for mycoplasma contamination. Cells were passaged at confluency, and ACLY deletion was confirmed by Western blotting after three passages. Pancreatic cancer ACLY KO cells were then transduced with a retroviral pOZ-N vector (Addgene, 3781) expressing ACLY or an empty pOZ-N vector to generate cells with reconstituted ACLY (ACLY KO plus ACLY cDNA) or an empty vector (ACLY KO). Cells were selected using interleukin-2R selection beads via pull down. To generate ACSS2 KO cells, cells were then transduced with a LentiCRISPR v2 empty vector or vector containing a guide RNA targeting the first exon of ACSS2. Following transduction, cells were treated with puromycin until the entirety of an untransduced cell population died. Following puromycin treatment, single-cell clonal populations were generated for ACLY KO cells with ACLY cDNA and ACSS2 KO as well as ACLY KO cells with ACSS2 KO by limiting serial dilution.

### Cell culture

Murine HCC cells were cultured in DMEM/F12 (Gibco, #11320033) supplemented with 10% super calf serum (FS) (Gemini, #100-510). Murine pancreatic cancer cells were cultured in DMEM (Gibco, #11965084) supplemented with 10% super calf serum (FS) (Gemini, #100-510). Cell growth experiments were performed by plating cells at the indicated density in DMEM/F12 + 10% FS, and cells were allowed to adhere overnight. Culture medium was changed the following day to the indicated conditions. Media for all experiments used high-glucose DMEM (Gibco, #11965084) or glucose- and glutamine-free DMEM (Gibco, #A1443001) supplemented with 10 mM glucose and 4 mM glutamine unless otherwise indicated. dFBS (Gemini, #100-108) and charcoal-stripped FBS (CDT; Corning, #35-072-CV or Sigma-Aldrich, F6765) were used when indicated. All cell lines were routinely tested for mycoplasma contamination.

Metabolite addback and inhibitor treatment experiments were performed in the media conditions listed with supplementation of the listed metabolite or drug with equal volumes of vehicle control unless otherwise specified. Acetate, pyruvate, and mevalonate were supplemented after dissolving in water. l-Carnitine and acetyl-l-carnitine were dissolved in media used for each experiment. Fatty acid supplementation used palmitate and/or oleate conjugated to fatty acid–free BSA (Bioworld, 22070023), and fatty acid–free BSA in an equivalent volume was supplemented as control.

### Soft agar colony formation assay

Cells were plated in six-well plates at a density of 2.5 × 10^4^ cells per well. First, plates were coated with glucose- and glutamine-free DMEM media containing 10 mM glucose and 4 mM glutamine and supplemented with 10% FS and 0.6% Bacto Agar. Cells were plated on top of the 0.6% Bacto agar layer in glucose- and glutamine-free DMEM media containing 10 mM glucose and 4 mM glutamine and supplemented with 10% FS and 0.3% Bacto Agar. Each cell line was plated in triplicate. Fresh medium was added to the wells every 7 days for 3 weeks. Images were taken for analysis after 3 weeks. A total of four non-overlapping images were taken of each well totaling 12 images for analysis per cell line. Colonies were counted after blinding of images.

### Acid extraction of histones

Acid extraction on isolated nuclei was performed as previously described (*4*). Cells were lysed with NIB-250 buffer [15 mM tris-HCl (pH 7.5), 60 mM KCl, 15 mM NaCl, 5 mM MgCl_2_, 1 mM CaCl_2_, 250 mM sucrose, 1 mM dithiothreitol, 10 mM sodium butyrate, and protease inhibitors] with 0.1% NP-40 for 5 min on ice. Nuclei were pelleted from the cell lysate by centrifugation at 600*g* at 4°C for 5 min. Extracted nuclei were washed with NIB-250 twice. Extracted nuclei were resuspended in 0.4 N of H_2_SO_4_ and rotated for 4 hours or overnight at 4°C to extract histone proteins. The extracts were cleared by centrifugation at 11,000*g* at 4°C for 10 min. Clarified histone extracts were precipitated by adding 100% trichloracetic acid to a final concentration of 20%, and the extracts were at 4°C overnight. Precipitated histones were centrifuged at 11,000*g* at 4°C for 10 min. Histones were washed with 1 ml of acetone + 0.1% 12 N of HCl once and 1 ml of acetone twice. The histone pellet was air-dried at room temperature and then resuspended in glass-distilled H_2_O. Resuspended histones were used for Western blotting.

### Western blotting

Cell for whole-cell protein lysates were collected by trypsin-mediated release from tissue culture plates. Cells were spun at 8000 rpm for 5 min and were kept on ice. Pellets were washed once with phosphate-buffered saline (PBS). The cell pellet was resuspended in 50 to 100 μl of radioimmunoprecipitation assay (RIPA) buffer [1% NP-40, 0.5% deoxycholate, 0.1% SDS, 150 mM NaCl, 50 mM tris plus protease inhibitor cocktail (Sigma-Aldrich P8340), and phosSTOP if phosphorylated proteins were being investigated (Sigma-Aldrich 04906845001)]. Cell lysis was allowed to occur on ice for 10 min. Cells were sonicated with a Branson Sonifier for 10 pulses at 20% amplitude. Cell lysate was clarified by centrifugation at 15,000*g* for 10 min at 4°C, and the supernatant was transferred to a new tube. Samples were stored at −80°C until analysis. All blots were developed using a LI-COR Odyssey CLx system. Antibodies used in this study were the following: ACLY (Proteintech, #15421-1-AP), ACSS2 (Cell Signaling Technology (CST), #3658), β-actin (CST, #3700), α-tubulin (CST, #2144), CrAT (Cloud-Clone Corp., #PAC400Mu01), PDHe1α (Santa Cruz Biotechnology, sc-377092), pan-acetyl-lysine (CST, #9441), acetyl-tubulin (CST, #3971), acetyl-H3K23 (CST, #14932), acetyl-H4 (Millipore, 06-866), acetyl-H3K27 (Abcam, ab4729), acetyl-H3K9 (Active Motif, AB_2793569), acetyl-H4K5 (Millipore, 07-327), fatty acid synthase (FASN; CST, #3189), HSP60 (CST, #12165), CS (CST, #14309), and VDAC (CST, #4661).

### Reverse transcription quantitative polymerase chain reaction

RNA was extracted from cells after trypsinization and pelleting by centrifugation at 8000*g* for 5 min. Pellets were resuspended in 500-μl TRIzol (Life Technologies). RNA was extracted following the TRIzol manufacturer protocol. Then, cDNA was prepared using high-capacity RNA-to-cDNA master mix (Applied Biosystems, 4368814) according to kit instructions. cDNA was diluted 1:20 and amplified with PowerUp SYBR Green Master Mix (Applied Biosystems, A25778) using a ViiA-7 real-time polymerase chain reaction system (Applied Biosystems). Fold change in expression was calculated by the ΔΔ*C*_t_ method using actin as a control. Primer sequences were as follows: mCrAT, TGGTCATCTACTCCAGCCCA (forward) and AACTGGCAGCGTCTCATTGT (reverse); mActin, TGGTGGGAATGGGTCAGAA (forward) and TCTCCATGTCGTCCCAGTTG (reverse).

### D_2_O and ^13^C-glucose labeling of fatty acids and FAME GC-MS

Cells were seeded at a density of 7.5 × 10^5^ cells per plate in DMEM/F12 containing 10% FS. For deuterium tracing, the following day, the media was changed to DMEM media containing 10% FS or 10% dFBS supplemented with 100 μM acetate and 10% deuterium oxide (Sigma-Aldrich, 151882). For glucose tracing, the following day, the media was changed to glucose- and glutamine-free DMEM media containing 10% dFBS or 10% CDT with 4 mM glutamine and 10 mM ^13^C_6_ glucose. After 24 or 48 hours, cells were washed with ice-cold Dulbecco's phosphate buffered saline and trypsinized. The trypsin reaction was stopped using cold 10% fatty acid–free BSA in DPBS to remove any exogenous fatty acids. The cells were then washed twice with ice-cold DPBS, and the cell pellet was frozen at −80°C until extraction.

Lipids were extracted by resuspending the cell pellet in 2-ml ice-cold methanol, followed by addition of 700-μl ice-cold glass-distilled water. Ten microliters of 1 mM heptadecanoic acid in methanol was added to each sample as an internal standard. Cell suspensions were sonicated using a Branson Sonifier 250 at an output of 2.5 and a duty cycle of 20% for 15 pulses. Following sonication, 1 ml of ice-cold chloroform was added, and the suspension was mixed by vortexing. An additional 700 μl of ice-cold chloroform and 700 μl of ice-cold glass-distilled water were added to the mixture and vortexed to mix. The suspension was centrifuged at 8000*g* for 10 min at 4°C. The chloroform fraction was transferred to a new tube, and the original suspension was reextracted with 700 μl of ice-cold chloroform and centrifuged at 8000*g* for 10 min at 4°C. The chloroform fraction from both extractions was pooled, 100 μl of ice-cold water was added, and the sample was vortexed to mix. The sample was centrifuged at 8000*g* for 10 min at 4°C, and the chloroform fraction was transferred to a new tube and dried under nitrogen at 40°C.

Lipids were derivatized to methyl-esters by first resuspending the dried lipid extracts in 2 ml of methanol:toluene (80:20) containing butylated-hydroxy toluene (5 mg in 50 ml). Acetyl-chloride (2 μl) was added, and the samples were heated to 95°C for 1 hour. Following heating, 5 ml of 6% potassium carbonate was added, and the samples were centrifuged at 8000*g* for 10 min at 4°C. The toluene layer was transferred to a new tube and centrifuged at 10,000 rpm for 5 min at room temperature. The toluene layer was transferred to a glass gas chromatography–mass spectrometry (GC-MS) vial with a volume-reducing insert. Fatty acid methyl esters were analyzed by GC-MS on an Agilent GC-MS 7890A/5975A with a DB-5 column. Deuterium or carbon enrichment into palmitate was determined using FluxFix (*54*).

### Acyl-CoA analysis by LC-MS

For extraction of acyl-CoAs, culture medium was completely aspirated from cells in 6-cm plates before adding 1 ml of ice-cold 10% trichloroacetic acid to plates. For quantification, experimental internal standard was added containing [^13^C_3_^15^N_1_]-labeled acyl-CoAs generated in pan6-deficient yeast culture (*55*). Plates were scraped to collect the cells. Samples were then sonicated for 10 × 0.5 s pulses to completely disrupt cellular membranes and incubated on ice to precipitate proteins. Protein was pelleted at 16,000*g* for 10 min at 4°C. The supernatant was collected and purified by solid-phase extraction using Oasis HLB 1cc (30 mg) solid phase extraction (SPE) columns (Waters). Eluate was evaporated to dryness under nitrogen gas and resuspended in 50 μl of 5% 5-sulfosalicylic acid (w/v) for injection. Samples were analyzed by an Ultimate 3000 autosampler coupled to a Thermo Q-Exactive Plus Instrument in positive electrospray ionization mode as previously described (*56*). For quantitation, a calibration curve was generated using commercially available standards, and internal standards containing [^13^C_3_^15^N_1_]-labeled acyl-CoAs generated in pan6-deficient yeast culture (*55*) were added to each sample. For enrichment analysis, isotopically labeled glucose (^13^C_6_ glucose), acetate (^13^C_2_ acetate), or palmitate (^13^C_16_ palmitate) was added to culture media, and the enrichment into acyl-CoAs was determined using FluxFix based on samples treated with no isotope tracer (*54*).

### Carnitine acyltransferase activity assay in whole-cell lysates

WT, ACLY KO, and ACLY KO CrAT KO cells were collected after trypsinization and counted via a Coulter counter. Cells were pelleted via centrifugation at 200*g* for 5 min at 4°C and washed twice with ice-cold 0.9% NaCl. Cell pellets were resuspended at 250,000 cells/ml in ice-cold lysis buffer [50 mM tris-HCl (pH 7.8) with 2 mM EDTA). One-milliliter aliquots of the cell suspensions were transferred to microfuge tubes and sonicated at 5 × 0.5 s pulses to lyse cells. Cell lysates were centrifuged at 17,000*g* for 10 min at 4°C to pellet cellular debris, and 20 μl of each clarified lysate was added to 80 μl of ice-cold methanol in separate wells of a 96-well plate (*t* = 0). To each well of a 96-well 2-ml deep-well plate, 400 μl of lysate was added, and reactions were initiated by the addition of 50 μl of 1 mM CoA and 50 μl of 50 mM acetylcarnitine. Negative control reactions were prepared using WT lysates that were boiled at 100°C for 10 min. At each time point, the reaction plate was vortexed briefly (~5 s) to mix, and 20 μl of each reaction mixture was added to 80 μl of ice-cold methanol in a 96-well plate on ice to quench the reaction and precipitate proteins. After the final time point, the plate was vortexed briefly (~5 s) to mix, followed by centrifugation at 2000*g* for 10 min to pellet proteins. For quantitation, calibration standards were prepared via serial dilution using commercially available acyl-CoA standards (Sigma-Aldrich), and 20 μl of each calibration standard was added to 80 μl of ice-cold methanol in a 96-well plate and processed in parallel with the samples. Clarified samples and standards were analyzed for acyl-CoAs via LC-MS as described above using 5-μl injections.

### Acyl-carnitine analysis by LC-MS

For extraction of acyl-carnitines, cells were washed with 5 ml of ice-cold 0.9% NaCl to remove extracellular metabolites and scraped on ice in 1-ml −80°C 80% high-performance liquid chromatography (HPLC)–grade methanol/20% HPLC-grade water. Extracts were collected in 1.5-ml tubes, and 10 ng of d3-propionyl-l-carnitine internal standard (Cayman, 26579) in 50 μl of 80% HPLC-grade methanol/20% HPLC-grade water was added to each sample. Samples were vortexed and incubated at −80°C for 30 min following centrifugation at 17,000*g* for 10 min at 4°C. Supernatants were transferred into a 96-well plate and dried under nitrogen gas at room temperature overnight. Dried metabolites were resuspended in 50 μl of 95% HPLC-grade water/ 5% HPLC-grade methanol using a Tomtec Quadra 4 . For each sample, 1 μl was injected and analyzed using a Vanquish Duo liquid chromatography (LC) system coupled to a Thermo Q-Exactive Plus Orbitrap Instrument in positive electrospray ionization mode in full-scan mode from 150 to 1000 mass/charge ratio. The LC system used a hydrophilic interaction chromatography analytical column (Ascentis Express, 2.1 mm by 150 mm, 2.7 μm). The column was kept at 30°C, and the flow rate was 0.5 ml/min. The mobile phase was solvent A (10 mM ammonium acetate and 0.2% formic acid in water) and solvent B (10 mM ammonium acetate and 0.2% formic acid in 95% HPLC-grade acetonitrile/5% HPLC-grade water). Elution gradients were run starting from 98% B to 86% B from 0 to 7 min; 86% B to 50% B from 7 to 7.3 min; 50% B to 10% B from 7.3 to 8.3; 10% B was held from 8.3 to 14.5 min, 10% B to 98% B from 14.5 to 14.510; 98% was held from 14.510 to 15 min, and then the column was equilibrated while eluting on the other identical column. For each analyte and the internal standard, the peak corresponding to the [M + H]^+^ ion at 5 ppm was integrated in TraceFinder 4.1 (Thermo Fisher Scientific). The enrichment of isotopically labeled glucose (^13^C_6_ glucose) or palmitate (^13^C_16_ palmitate) into acetyl-carnitine was determined using FluxFix based on samples treated with no isotope tracer (*54*).

### TCA metabolite analysis by GC-MS

Polar metabolites were extracted from cells through addition of 1 ml of 80:20 methanol:water chilled to −80°C. Cells were collected by scraping after addition of norvaline as an internal standard, lysed by three rounds of freeze thawing, and insoluble material was removed through centrifugation at 12,000*g* at 4°C for 10 min. The pellet was used for protein quantification after resuspension in 2% SDS 0.1 mM tris buffer. The supernatant containing polar metabolites was evaporated to dryness by SpeedVac. The dried pellet was stored at −80°C until derivatization. Samples were derivatized by addition of 30 μl of methoxyamine (5 mg/ml) in pyridine and heated for 15 min at 70°C. A total of 70 μl of N-tert-Butyldimethylsilyl-N-methyltrifluoroacetamide (MTBSTFA) was then added, and the samples were heated at 70°C for 1 hour. After derivatization, samples were centrifuged at 12,000 rpm for 5 min. The supernatant was transferred to a glass GC-MS vial with avolume-reducing insert. Samples were analyzed by GC-MS on an Agilent GC-MS 7890A/5975A with a DB-5 column. 13-C enrichment was determined using FluxFix based on samples treated with no isotope tracer (*54*). Relative quantification was performed by normalizing the sum of the area under the curve (AUC) of all isotopologues for each metabolite to the sum of the AUC of all isotopologues of norvaline within each sample followed by normalization to the average protein quantification within each experimental group.

### Histone acetylation UPLC-MS/MS analysis

Histones were extracted as described in the “Acid extraction of histones” section. Dry histone pellets were stored at −80°C until processing for LC/MS analysis. The unmodified lysines in dry histone were propionylated by propionic anhydride at pH 8 and 51°C for 1 hour followed by trypsin digestion at pH 8 and 37°C for overnight as in our previously published procedures (*57*). A Waters Acquity H-class Ultra-performance liquid chromatography (UPLC) coupled with a Thermo TSQ Quantum Access triple quadrupole mass spectrometer was used to quantify the acetylated lysines on H3 tryptic peptides. The UPLC and tandem mass spectrometry (MS/MS) settings, solvent gradient, and detailed mass transitions were reported previously (*58*). Retention time and specific mass transitions were both used to identify individual acetylated and propionylated peaks. The ^13^C-labeled acetyl-lysine peptide peaks were identified by considering the shift in mass by +2. The resolved peaks were integrated using Xcalibur software (version 2.1, Thermo Fisher Scientific). Relative quantitative analysis was used to determine the amount of modification on individual lysines.

### SILEC-SF acyl-CoA quantitation

SILEC-SF was performed as previously described (*22*). Briefly, WT HCC cells (D42) were used to generate SILEC internal standard by passaging of cells in ^15^N^13^C_3_-pantothenate (vitamin B5) for at least nine passages as previously described (*22*). SILEC WT cells were mixed with DKO1 cells before fractionation. Mitochondria and cytosolic fractions were separated through differential centrifugation. Briefly, cells were scraped in mannitol-sucrose buffer containing 210 mM mannitol, 70 mM sucrose, 5 mM tris-HCl (pH 7.5), and 1 mM EDTA (pH 7.5). After scraping, cells were homogenized in a glass vessel with ceramic homogenizer at 1600 rpm. An aliquot of the cell lysate was taken as the whole-cell lysate (WCL). Lysates were spun at 1300*g* for 10 min at 4°C. The pellet was in trichloroacetic acid for debris. The supernatant was spun at 10,000*g* for 20 min at 4°C. The pellet was resuspended in trichloroacetic acid for mitochondria. The supernatant was added to trichloroacetic acid to a final concentration of 10% for cytosol. Acyl-CoAs were extracted as above and were subjected to analysis by LC-MS as described above.

### RNA sequencing

RNA was extracted from HCC cells cultured in DMEM + 10% FS for 24 hours using a Qiagen RNeasy Plus Mini Kit (Qiagen, #74134) with an additional on column DNA digestion using Qiagen TURBO deoxyribonuclease (Qiagen, #AM2238) based on the manufacturer’s protocols.

RNA-seq libraries were prepared with the NEBNext poly(A) Magnetic Isolation Module (New England Biolabs (NEB), #E7490L) followed by the NEBNext Ultra Directional RNA library preparation kit for Illumina (NEB, #E7420L) according to the manufacturer’s protocol. Library quality was assessed using an Agilent BioAnalyzer 2100, and libraries were quantified with the Library Quant Kit for Illumina (NEB, #E7630L). Libraries were then diluted to 1.8 pM and sequenced on the NextSeq 500 platform using 75–base pair single-end reads. All RNA-seq read alignment was performed using Illumina RNA-seq alignment software (version 2.0.1). Briefly, reads were mapped to *Mus musculus* University of California Santa Cruz (UCSC) mouse GRCm38/mm10 reference genome with the RNA STAR aligner under default settings (version 2.6.1a) (*59*). Transcripts per million generation and differential expression analysis were performed on aligned reads to *M. musculus* UCSC GRCm38/mm10 reference genome using Illumina RNA-seq differential expression software (DESeq2, v1.0.1) (*60*). Significance cutoffs are listed in figure legends for each analysis. GSEA (*61*, *62*) was performed by comparing DKO cells to all other genotypes combined (WT, ACLY KO, and ACSS2 KO). RNA-seq data are available on Gene Expression Omnibus (GEO; GSE223966).

### Immunofluorescence confocal microscopy

Cells were plated on glass coverslips and allowed to adhere for 24 hours. Live cells were then incubated in DMEM + 10% FS with 1 μM MitoTracker Deep Red FM (Thermo Fisher Scientific) for 30 min. Cells were washed three times with PBS and then fixed with 4% paraformaldehyde for 30 min. After fixation, coverslips were washed three times with Tris buffered saline with tween (TBST) and then blocked with 10% goat serum, 1% BSA, 0.1% gelatin, glycine (22.52 mg/ml), and 0.1% Triton X-100 in TBST for 30 min. Coverslips were washed three times with TBST and then incubated with PDHe1α antibody (Abcam, ab110334) for 3 hours. Coverslips were washed three times with TBST and then incubated with 4′,6-diamidino-2-phenylindole and donkey anti-rabbit Alexa Flour 488 (Invitrogen, #a21206) for 1 hour. Coverslips were washed three times and then mounted on slides for imaging. Slides were imaged on a Zeiss LSM 880 confocal microscope. Z-stacks were compressed into a single plane for representation.

### Mitochondrial fractionation

Mitochondrial fractionation was performed by scraping cells in mannitol-sucrose buffer containing 210 mM mannitol, 70 mM sucrose, 5 mM tris-HCl (pH 7.5), and 1 mM EDTA (pH 7.5). After scraping, cells were homogenized in a glass vessel with ceramic homogenizer at 1600 rpm. An aliquot of the sample was taken after homogenization and was diluted 1:1 in RIPA for WCL. The remaining homogenized cell lysate was spun at 1300*g* for 10 min at 4°C. The pellet was resuspended in RIPA for debris. The supernatant was spun at 10,000*g* for 20 min at 4°C. The pellet was resuspended in RIPA for mitochondria. The supernatant was clarified for cytosol. Protein samples were quantified, and Western blotting was performed using the method above.

### Statistical analyses

All analyses were performed using GraphPad Prism or R (RNA-seq analysis). Statistical test specifics are included in figure legends. Comparisons without significance are not included in graphs. Multiple comparisons were made between groups against the control condition unless otherwise stated. All experiments are representative of at least two independent replicates except for SILEC-SF experiments, histone post-translational modification (PTM) analysis by LC-MS, RNA-seq, and fatty acid tracing into acetyl-CoA with etomoxir and thioridazine treatment.

## Notes

View/request a protocol for this paper from *Bio-protocol*.
