# Supplementary material for: Acetylcarnitine shuttling links mitochondrial metabolism to histone acetylation and lipogenesis
Source: Sci Adv. 2023 May 3;9(18):eadf0115. doi: 10.1126/sciadv.adf0115 (PMC10156126; doi:10.1126/sciadv.adf0115)
Supplement: Supplementary file 1 — Figs. S1 to S8 Table S1 [file sciadv.adf0115_sm.pdf]

Science Advances
AAAS

Supplementary Materials for

Acetylcarnitine shuttling links mitochondrial metabolism to histone acetylation and lipogenesis

Luke T. Izzo *et al.*

Corresponding author: Kathryn E. Wellen, wellenk@upenn.edu; Nathaniel W. Snyder, natewsnyder@temple.edu

Sci. Adv. **9**, eadf0115 (2023)
DOI: 10.1126/sciadv.adf0115

This PDF file includes:

Figs. S1 to S8
Table S1

Supplemental Figure 1: ACLY KO cells remain viable without acetate
A) Western blot for ACLY and ACSS2 in WT, ACLY KO, and ACSS2 KO HCC cells.
B) Representative images of soft agar colony formation assay for WT, ACLY KO, and ACSS2 KO HCC cells.
C) Representative images of ACLY KO MEFs (PC9 cells) and ACLY KO HCC cells grown in the absence of acetate for 0 hours and 96 hours. Cells were plated near confluency and monitored for cell death through decreasing confluency over time.
D) Pancreatic cancer ACLY KO cell proliferation in DMEM + 10% FS, DMEM + 10% dFBS, or DMEM + 10% dFBS + 100µM acetate for 72 hours. Statistical significance was calculated by one-way ANOVA
Each point represents a biological replicate and error bars represent standard deviation. *$p \leq 0.05$; **$p \leq 0.01$; ***$p \leq 0.001$; ****$p \leq 0.0001$

Supplemental Figure 2: DKO cancer cells can proliferate and maintain acetyl-CoA pools.
A) Western blot for ACLY and ACSS2 in Pancreatic cancer ACLY KO cell lines expressing ACLY cDNA or lacking ACSS2 after CRISPR/Cas9 mediated knockout.
B) Proliferation of pancreatic cancer cell lines over 5 days in DMEM + 10% FS. Statistical significance was calculated by one-way ANOVA.
C) Western blot for ACLY and ACSS2 in HCC cells cultured in 10% FS at atmospheric oxygen (-) or 1% FS in 1% oxygen (+) for 24 hours to stimulate ACSS2 expression. S.E. short exposure; L.E. long exposure.
D) Whole cell acetyl-CoA measurements in HCC cells cultured in DMEM + 10% FS. Statistical significance was calculated by one-way ANOVA.
E) Acyl-CoA quantitation using SILEC-subcellular fractionation performed on ACLY KO and DKO1 cells grown in DMEM + 10% FS for 24 hours. Acyl-CoA abundance can be compared within but not between compartments.
Each point represents a biological replicate and error bars represent standard deviation. *$p \leq 0.05$; **$p \leq 0.01$; ***$p \leq 0.001$; ****$p \leq 0.0001$

Supplemental Figure 3: Fatty acid metabolism is altered in DKO cells.
A) Principal component analysis of log2 transformed DESeq counts from RNAseq performed on cells grown in DMEM + 10% FS.
B) Volcano plots showing differentially expressed genes comparing KO genotypes to WT cells. Dots represent individual genes. Red dots are genes with a log2 fold change>1.5 and adjusted p-value<0.01.
C) Venn diagrams comparing the differentially expressed genes (log2 fold change>1.5 and adjusted p-value<0.01) between each KO genotype and WT cells.
D) Heatmap of all genes from the hallmarks fatty acid metabolism gene set. DESeq counts were log2 transformed before clustering. Red box shows the gene cluster used to generate the graph in figure 3C.
E) Cell proliferation after 96 hours. Cells were plated in DMEM/F12 media overnight then cultured in DMEM + 10% FS or CDT serum with or without the addition of metabolites. PA/OA is 100 µM of each fatty acid conjugated to BSA (200 µM total). Statistical significance was calculated by two-way ANOVA.

F) Cell proliferation after 96 hours. Cells were plated in DMEM/F12 media for overnight then cultured in DMEM + 10% FS or CDT serum with or without the addition of 100 μM of each fatty acid conjugated to BSA. Statistical significance was calculated by one-way ANOVA.
G) Cell proliferation after 96 hours. Cells were plated in DMEM/F12 media overnight then cultured in DMEM + 10% FS or dFBS serum with or without the addition of metabolites. PA/OA is 100 μM of each fatty acid conjugated to BSA (200 μM total). Statistical significance was calculated by two-way ANOVA.
H) Isotopologue enrichment of palmitate measured by GC-MS. Cells were cultured in DMEM + 10% D2O + 10% FS for 24 hours. Statistical analysis performed on total hydrogen enrichment. Statistical significance was calculated by one-way ANOVA. No significance was found.
Each point represents a biological replicate and error bars represent standard deviation. *$p \leq 0.05$; **$p \leq 0.01$; ***$p \leq 0.001$; ****$p \leq 0.0001$

Supplemental Figure 4: Regulation of acetylation by fatty acids may act in part through acetyl-CoA sparing by ACC inhibition
A) Whole cell protein extract western blot from cells grown in DMEM + 10% FS and treated with 500 nM TSA and 500 μM nicotinamide (NAM) over a time course.
B) Acid extracted histone western blot from cells cultured in DMEM + 10% CDT and supplemented with PA/OA or 5mM octanoate. PA/OA is 100 or 500 μM of each fatty acid conjugated to BSA. Ponceau S stain for total protein in histone extracts used for western blot.
C) Whole cell acetyl-CoA quantitation of cells cultured in DMEM + 10% CDT with or without the addition PA/OA. PA/OA is 100 μM of each fatty acid conjugated to BSA (200 μM total). Statistical significance was calculated by multiple t-tests.
D) Whole cell malonyl-CoA quantitation of cells cultured in DMEM + 10% CDT with or without the addition PA/OA or the ACC inhibitor ND630. PA/OA is 100 μM of each fatty acid conjugated to BSA (200 μM total). Statistical significance was calculated by two-way ANOVA.
E) Acid extracted histone western blot from cells cultured in DMEM + 10% CDT or DMEM + 10% dFBS with or without ND630. Ponceau S stain for total protein in histone extracts used for western blot.
F) Acid extracted histone western blot from cells cultured in DMEM + 10% FS and octanoate for 24 hours.
G) Whole cell acetyl-CoA quantitation of WT and DKO cells cultured in DMEM + 10% FS supplemented with octanoate for 24 hours. Statistical significance was calculated by two-way ANOVA.
Each point represents a biological replicate and error bars represent standard deviation. *$p \leq 0.05$; **$p \leq 0.01$; ***$p \leq 0.001$; ****$p \leq 0.0001$

Supplemental Figure 5: Glucose and fatty acid contribution to histone acetylation may be through parallel or convergent pathways
A) $^{13}C_{16}$-palmitate tracing into acetyl-CoA analyzed by LC-MS. Cells were cultured in glucose and glutamine free DMEM + 10% CDT supplemented with 4 mM glutamine, 10 mM glucose and 100 μM $^{13}C_{16}$-palmitate conjugated to BSA for 2, 6, or 24 hours.
B) Whole cell acetyl-CoA quantitation in cells grown in glucose and glutamine free DMEM + 10% CDT supplemented with 4 mM glutamine and either 10 mM $^{13}C_6$-glucose and 100 μM palmitate conjugated to BSA or 10 mM glucose and 100 μM $^{13}C_{16}$-palmitate conjugated to BSA for 2 hours. Statistical significance was calculated by one-way ANOVA.
C) Whole cell acetyl-CoA quantitation in cells grown in glucose and glutamine free DMEM + 10% dFBS supplemented with 4 mM glutamine and 10 mM glucose and 100 μM acetate for 6 hours. Statistical significance was calculated by one-way ANOVA.

D) Total of all acetylation measured on acid extracted histone coming from $^{13}C_6$-glucose (Glc) or $^{13}C_{16}$-palmitate (PA) after 24 hours or incubation. Unlabeled acetylation marks are shown as "other sources".
E) Schematic showing potential explanations for glucose and palmitate labeling into acetyl-groups in the nuclear compartment. Top half represents a route for both palmitate and glucose to feed into a precursor acetyl-CoA pool in the mitochondria, bottom half represents two potential separate pathways involving peroxisomal beta-oxidation of fatty acids and pyruvate conversion to acetyl-CoA outside of the mitochondria. Created with BioRender.com.
F) TCA cycle intermediate relative quantitation. Relative abundance was normalized to protein content. Statistical significance was calculated by one-way ANOVA.
G) $^{13}C_{16}$-palmitate tracing into acetyl-CoA analyzed by LC-MS. Cells were cultured in glucose and glutamine free DMEM + 10% CDT supplemented with 4 mM glutamine, 10 mM glucose and 100 µM $^{13}C_{16}$-palmitate conjugated to BSA for 2 hours. Prior to labeling, cells were pre-treated for 15 minutes with the CPT1 inhibitor etomoxir (20 µM Eto), the peroxisomal beta-oxidation inhibitor thioridazine (10 µM Thio) or a combination of both drugs (Combo), and inhibitors remained on cells during labeling. Statistical significance was calculated by one-way ANOVA.
H) Acid extracted histone western blot from cells cultured in DMEM + 10% CDT. Cells were treated for with the CPT1 inhibitor etomoxir (Eto), the peroxisomal beta-oxidation inhibitor thioridazine (Thio) or a combination of both drugs. PA/OA is 100 µM of each fatty acid conjugated to BSA (200 µM total). Ponceau S stain for total protein in histone extracts used for western blot.
Each point represents a biological replicate and error bars represent standard deviation. *$p \leq 0.05$; **$p \leq 0.01$; ***$p \leq 0.001$; ****$p \leq 0.0001$

Supplemental Figure 6: PDH co-localizes with the mitochondria and is not regulated at the protein level by ACLY, ACSS2, or fatty acids
A) Confocal microscopy images of HCC cells cultured in DMEM + 10% FS for 24 hours. Scale bar is 25 µM.
B) Enlarged images from dotted inserts in panel A.
B) Western blot for PDHe1α in whole cell lysates from HCC cells cultured in DMEM + 10% FS for 24 hours.
C) Western blot for PDHe1α in whole cell lysates from HCC cells cultured in DMEM + 10% CDT with or without the addition PA/OA for 24 hours. PA/OA is 100 µM of each fatty acid conjugated to BSA (200 µM total).

Supplemental Figure 7: Acetylcarnitine shuttling increases glucose derived de novo lipogenesis in the absence of ACLY
A) Deuterium tracing into palmitate in DKO1 cells measured by GC-MS. Cells were cultured in DMEM + 10% D_2O + 10% CDT, +/- 10 mM carnitine for 24 hours. Statistical significance was calculated by unpaired t-tests.
B) Deuterium tracing into stearate in DKO1 cells measured by GC-MS. Cells were cultured in DMEM + 10% D_2O + 10% CDT, +/- 10 mM carnitine for 24 hours. Statistical significance was calculated by unpaired t-tests.
C) $^{13}C_6$-glucose tracing into stearate measured by GC-MS. Cells were cultured in glucose and glutamine free DMEM + 10% CDT supplemented with 4 mM glutamine and 10 mM $^{13}C_6$-glucose, +/- 10 mM carnitine for 48 hours. Statistical significance was calculated by unpaired t-tests.
D) Schematic showing glucose labeling into fatty acids through the proposed carnitine shuttle and the impact of addition of exogenous carnitine or acetylcarnitine to cells. Created with BioRender.com.

E) $^{13}C_6$-glucose tracing into stearate measured by GC-MS. Cells were cultured in glucose and glutamine free DMEM + 10% dFBS supplemented with 4 mM glutamine and 10 mM $^{13}C_6$-glucose with or without 10 mM carnitine or 10 mM acetylcarnitine for 48 hours.
F) Full isotopologue distribution of $^{13}C_6$-glucose tracing into palmitate measured by GC-MS. Cells were cultured in glucose and glutamine free DMEM + 10% dFBS supplemented with 4 mM glutamine and 10 mM $^{13}C_6$-glucose with or without 10 mM carnitine or 10 mM acetylcarnitine for 48 hours.
G) $^{13}C_6$-glucose tracing into palmitate and stearate measured by GC-MS in pancreatic cancer cells. Cells were cultured in glucose and glutamine free DMEM + 10% dFBS supplemented with 4 mM glutamine and 10 mM $^{13}C_6$-glucose with or without 10 mM carnitine for 48 hours. Statistical significance was calculated by unpaired t-tests.
H) Palmitate and stearate quantification in DKO1 cells. Cells were cultured in glucose and glutamine free DMEM + 10% CDT supplemented with 4 mM glutamine and 10 mM glucose with or without 10 mM carnitine or acetylcarnitine for 48 hours. Abundance was normalized to cell number. Statistical significance was calculated by one-way ANOVA.
I) Palmitate quantification in WT and ACLY KO cells. Cells were cultured in glucose and glutamine free DMEM + 10% dFBS supplemented with 4 mM glutamine and 10 mM glucose with or without 10 mM carnitine or acetylcarnitine for 48 hours. Abundance was normalized to cell number. Statistical significance was calculated by two-way ANOVA.
J) $^{13}C_6$-glucose tracing into acetyl-CoA, analyzed by LC-MS. Cells were cultured in glucose and glutamine free DMEM + 10% dFBS supplemented with 4 mM glutamine and 10 mM $^{13}C_6$-glucose supplemented with or without 10 mM carnitine or 10 mM acetylcarnitine for 6 hours. Statistical significance was calculated by two-way ANOVA.
K) $^{13}C_6$-glucose tracing into stearate measured by GC-MS after esterification of fatty acids. Cells were cultured in glucose and glutamine free DMEM + 10% dFBS supplemented with 4 mM glutamine and 10 mM $^{13}C_6$-glucose with 10 mM carnitine with vehicle control or 20 µM UK5099 for 48 hours. Statistical significance was calculated by unpaired t-tests.
L) Representative images of ACLY KO cells grown in glucose and glutamine free DMEM + 10% dFBS supplemented with 4 mM glutamine and 10 mM $^{13}C_6$-glucose with 10 mM carnitine with vehicle control or 20 µM UK5099 for 48 hours.
Each point represents a biological replicate and error bars represent standard deviation. *$p \leq 0.05$; **$p \leq 0.01$; ***$p \leq 0.001$; ****$p \leq 0.0001$

Supplemental Figure 8: CrAT supports acetyl-unit shuttling but is not required for acetylcarnitine to acetyl-CoA conversion
A) RT-qPCR analysis of CrAT KO clones. ΔΔCt analysis was performed using actin as the internal control gene and ACLY KO cells as the control condition.
B) Western blot for CrAT in whole cell lysates from ACLY KO and ACLY KO CrAT KO cells cultured in DMEM + 10% FS for 24 hours.
C) Western blot for CrAT in whole cell lysates of WT and CrAT KO cells cultured in DMEM + 10% FS for 24 hours.
D) Cell proliferation after 96 hours. Cells were plated in DMEM/F12 media overnight then cultured in DMEM + 10% FS. Statistical significance was calculated by one-way ANOVA.
E) Cell proliferation after 96 hours. Cells were plated in DMEM/F12 media overnight then cultured in DMEM + 10% FS. Statistical significance was calculated by one-way ANOVA.
F) $^{13}C_6$-glucose tracing into palmitate and stearate measured by GC-MS. Cells were cultured in glucose and glutamine free DMEM + 10% dFBS supplemented with 4 mM glutamine and 10 mM $^{13}C_6$-glucose with or without 10 mM carnitine for 48 hours.
G) $^{13}C_{16}$-palmitate tracing into acetylcarnitine, analyzed by LC-MS. Cells were cultured in glucose and glutamine free DMEM + 10% CDT supplemented with 4 mM glutamine, 10 mM

glucose, and 100 μM $^{13}C_{16}$-palmitate conjugated to BSA for 6 hours. Statistical significance was calculated by two-way ANOVA.
H) Mitochondrial fractionation western blot of ACLY KO CrAT KO cell lines.
I) Acid extracted histone western blot from cells cultured in DMEM + 10% dFBS supplemented with or without acetate, acetylcarnitine, or the ACSS2 inhibitor VY-3-135 for 24 hours.
J) Deuterium tracing into stearate measured by GC-MS. Cells were cultured in DMEM + 10% D_2O + 10% dFBS, +/- 10 mM acetate, carnitine or acetylcarnitine for 24 hours. Statistical significance was calculated by two-way ANOVA.
Each point represents a biological replicate and error bars represent standard deviation. *$p \leq 0.05$; **$p \leq 0.01$; ***$p \leq 0.001$; ****$p \leq 0.0001$

Supplemental Table 1: Differentially regulated genes from cluster analysis in Figure 3A

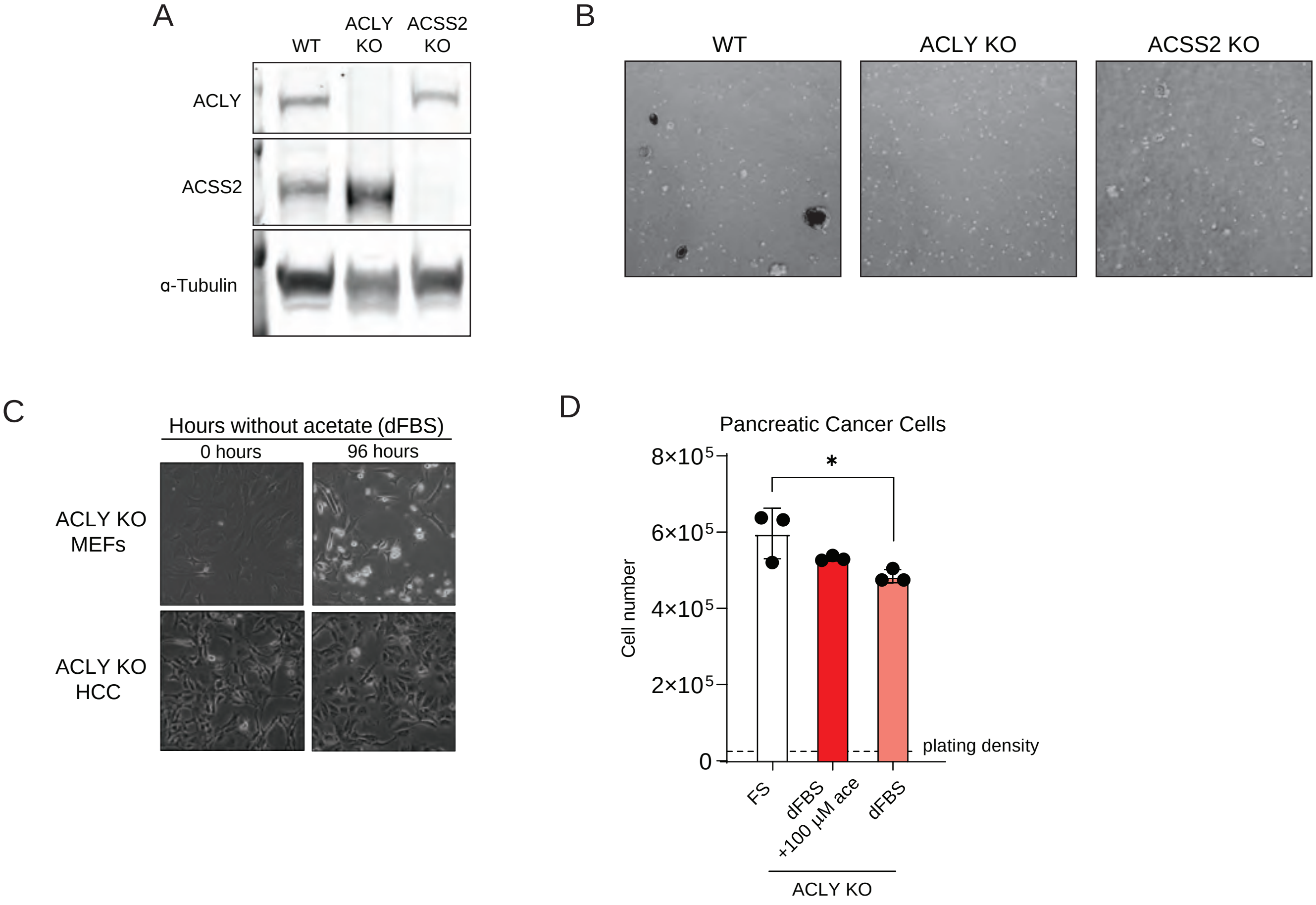

Supplemental Figure 1: ACLY KO cells remain viable without acetate

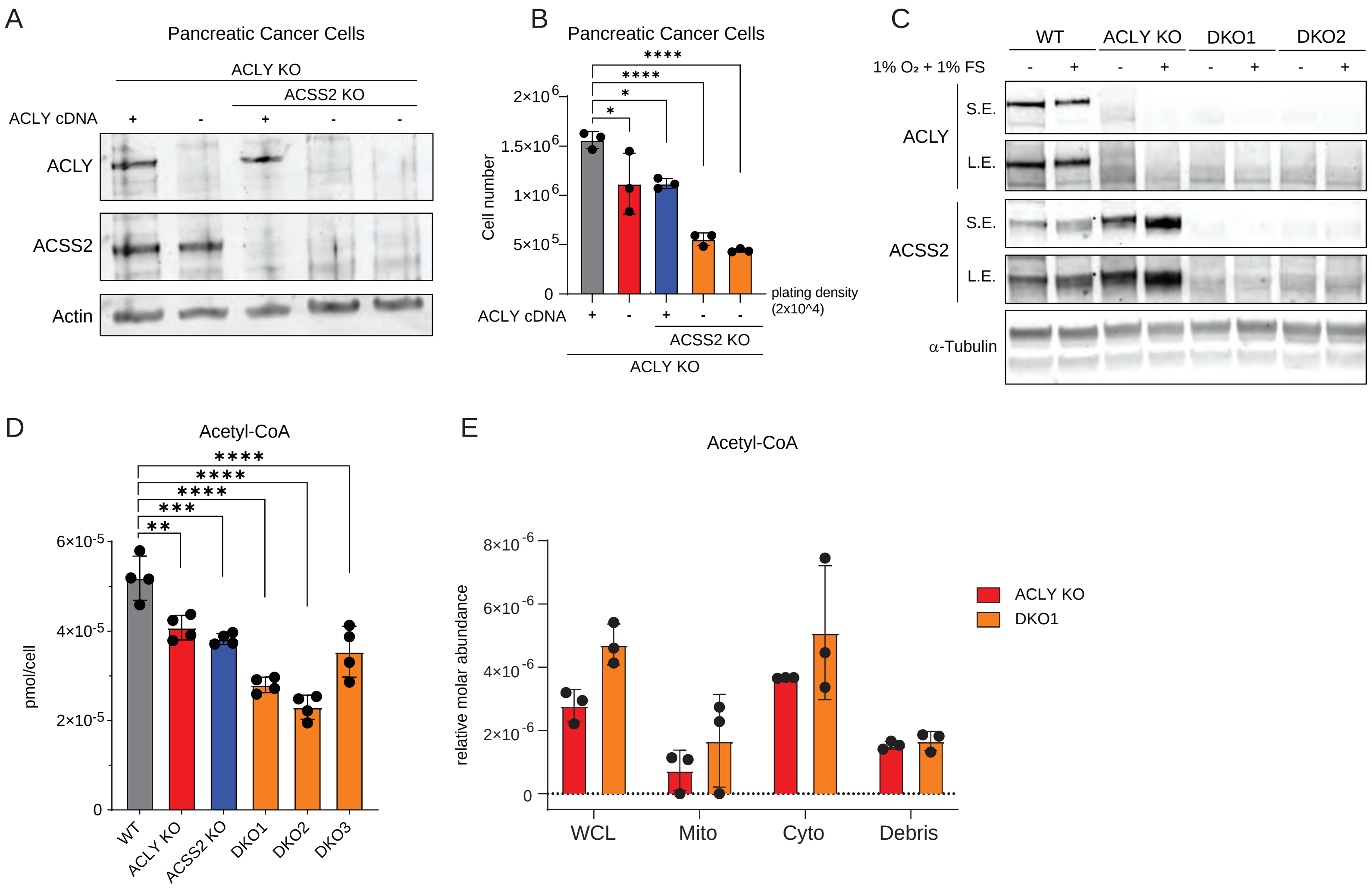

Supplemental Figure 2: DKO cancer cells can proliferate and maintain acetyl-CoA pools

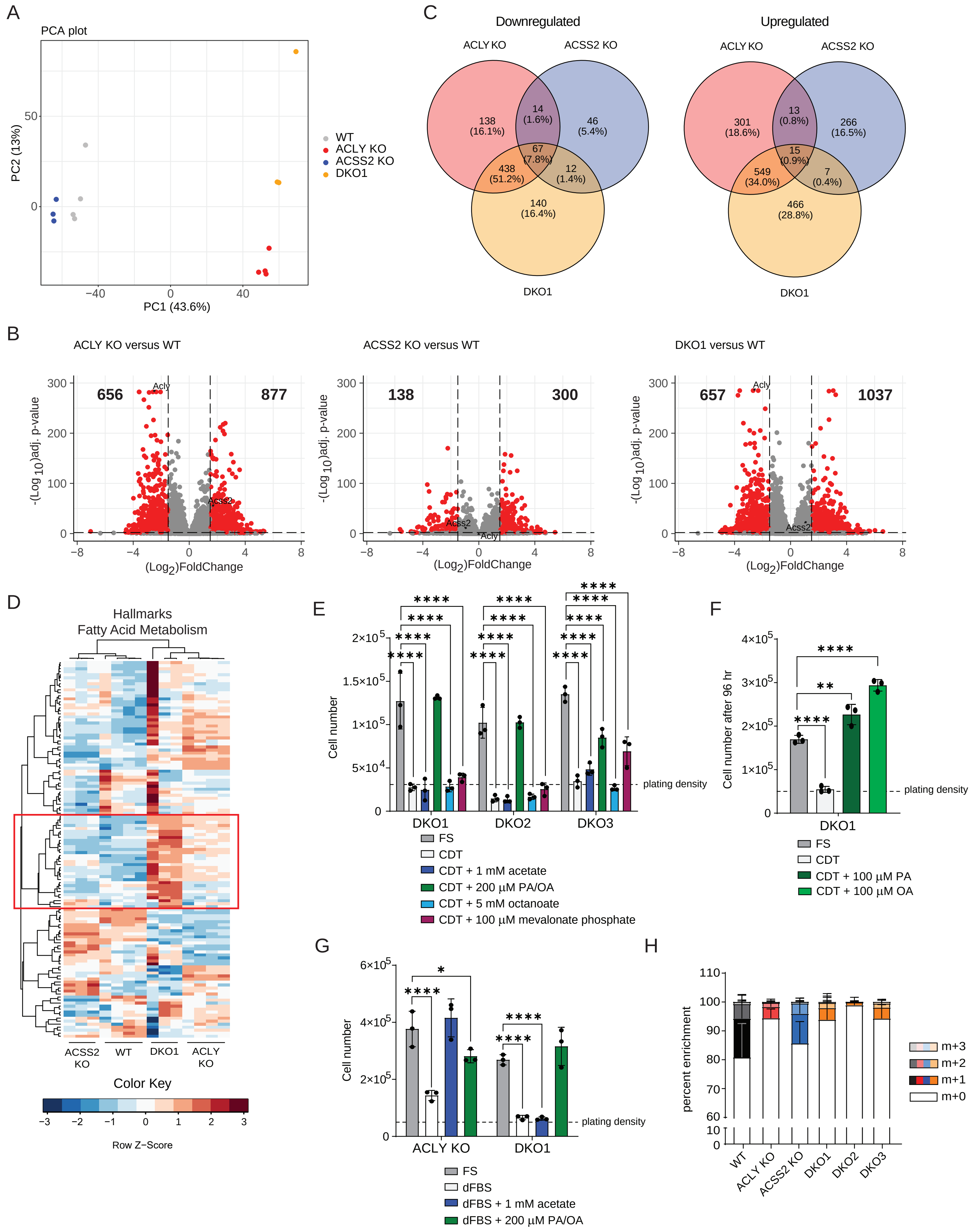

Supplemental Figure 3: Fatty acid metabolism is altered in DKO cells.

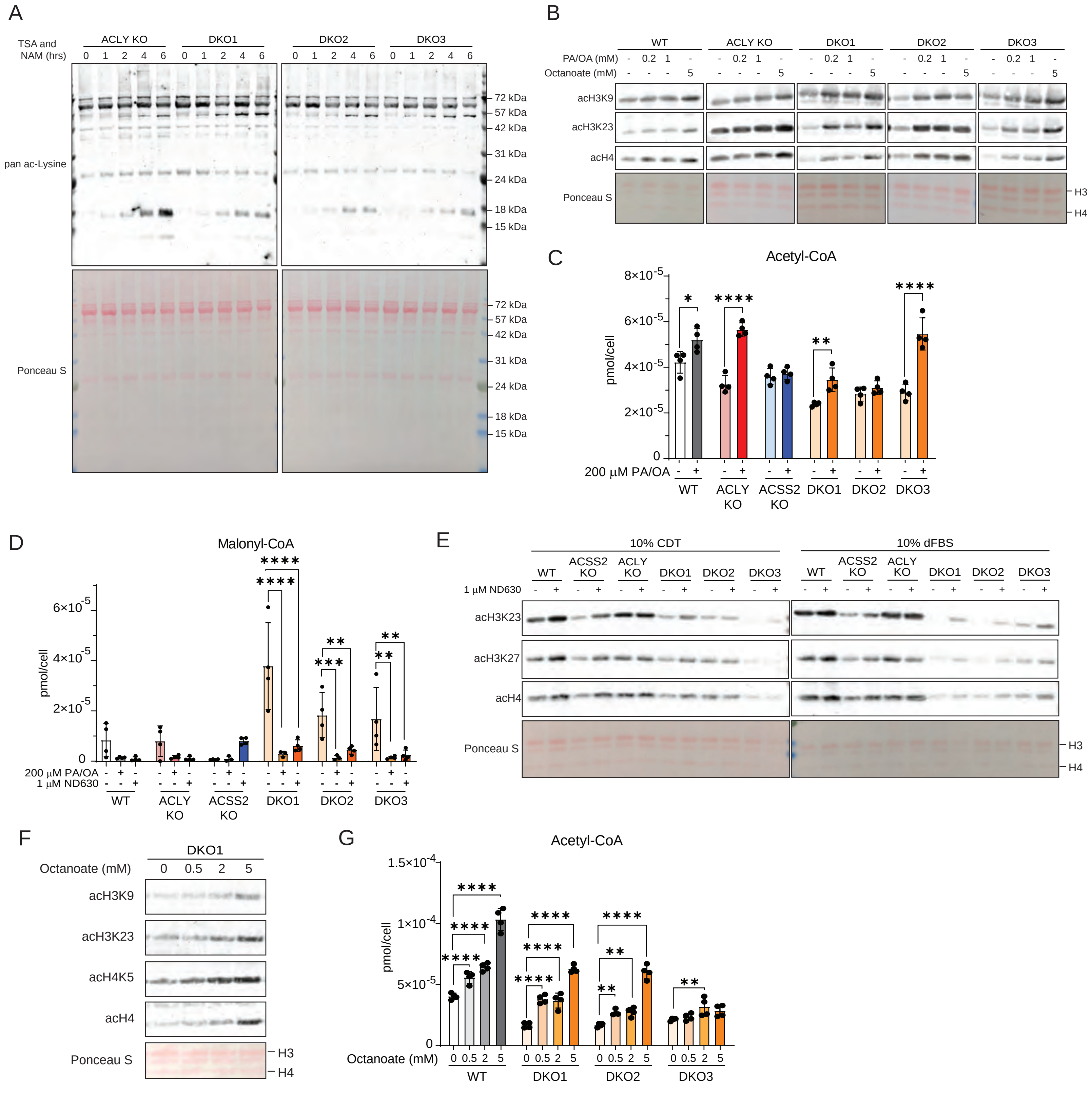

Supplemental Figure 4: Regulation of acetylation by fatty acids may act in part through acetyl-CoA sparing by ACC inhibition

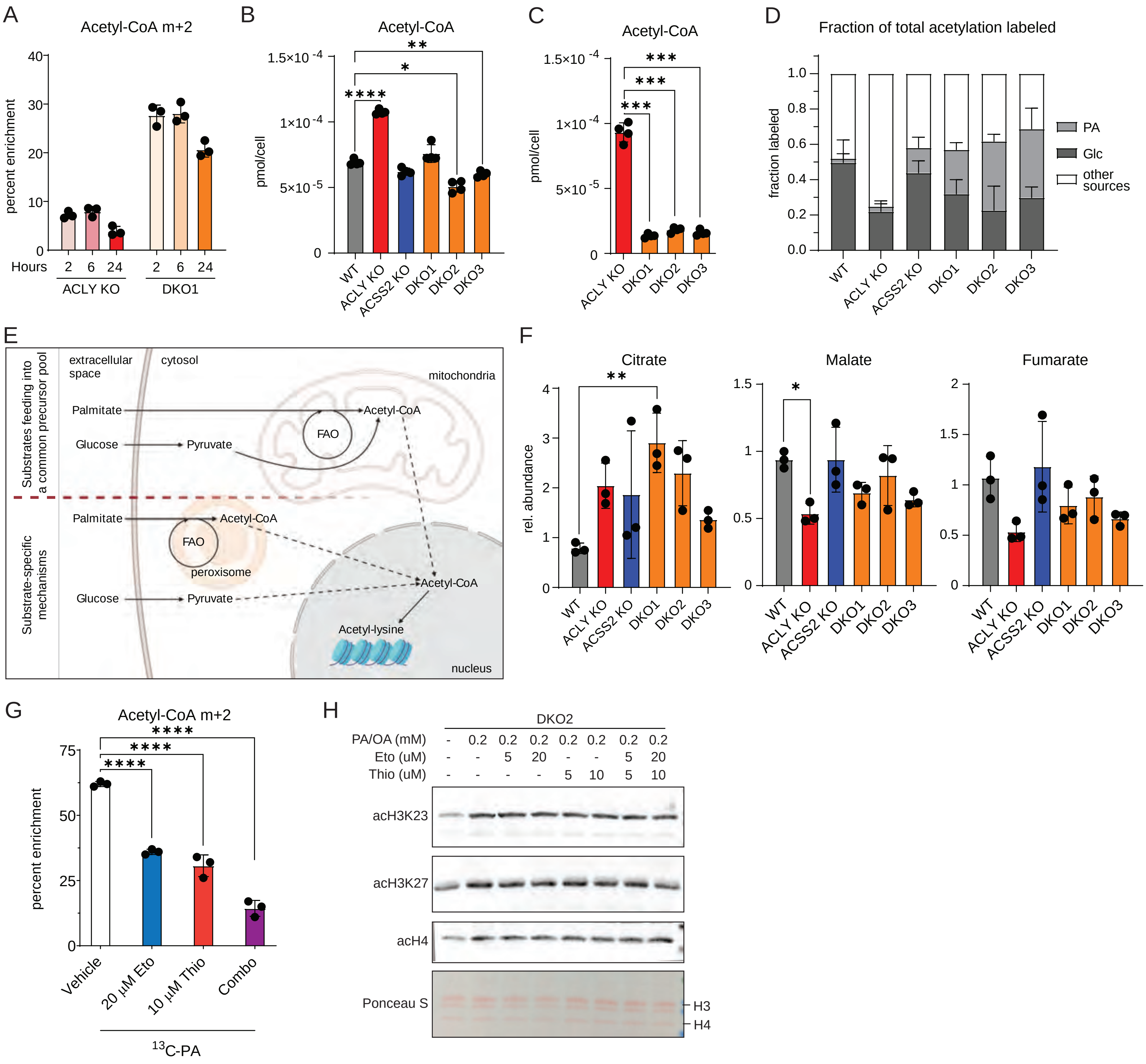

Supplemental Figure 5: Glucose and fatty acid contribution to histone acetylation may be through parallel or convergent pathways

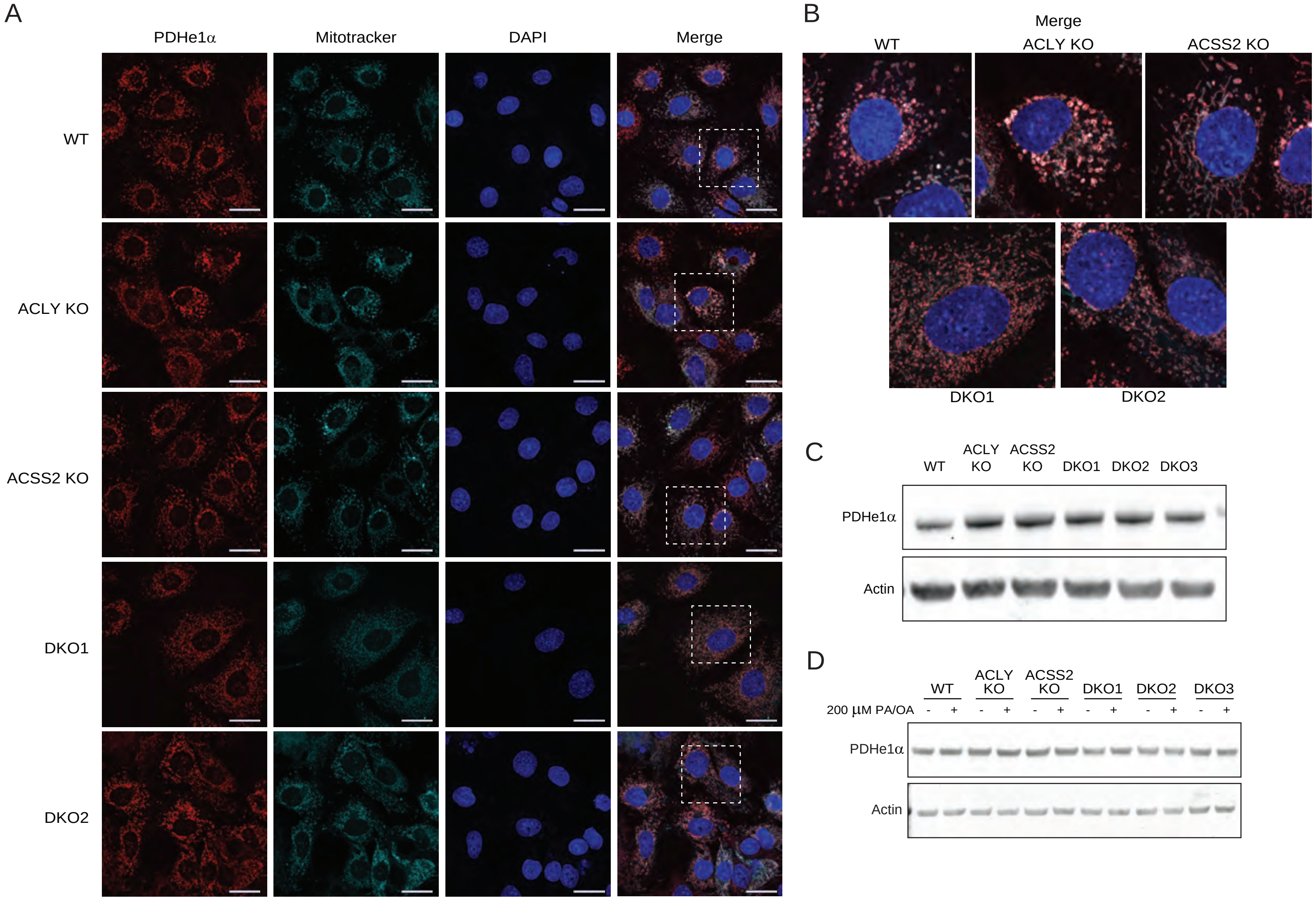

Supplemental Figure 6: PDH co-localizes with the mitochondria and is not regulated at the protein level by ACLY, ACSS2, or fatty acids

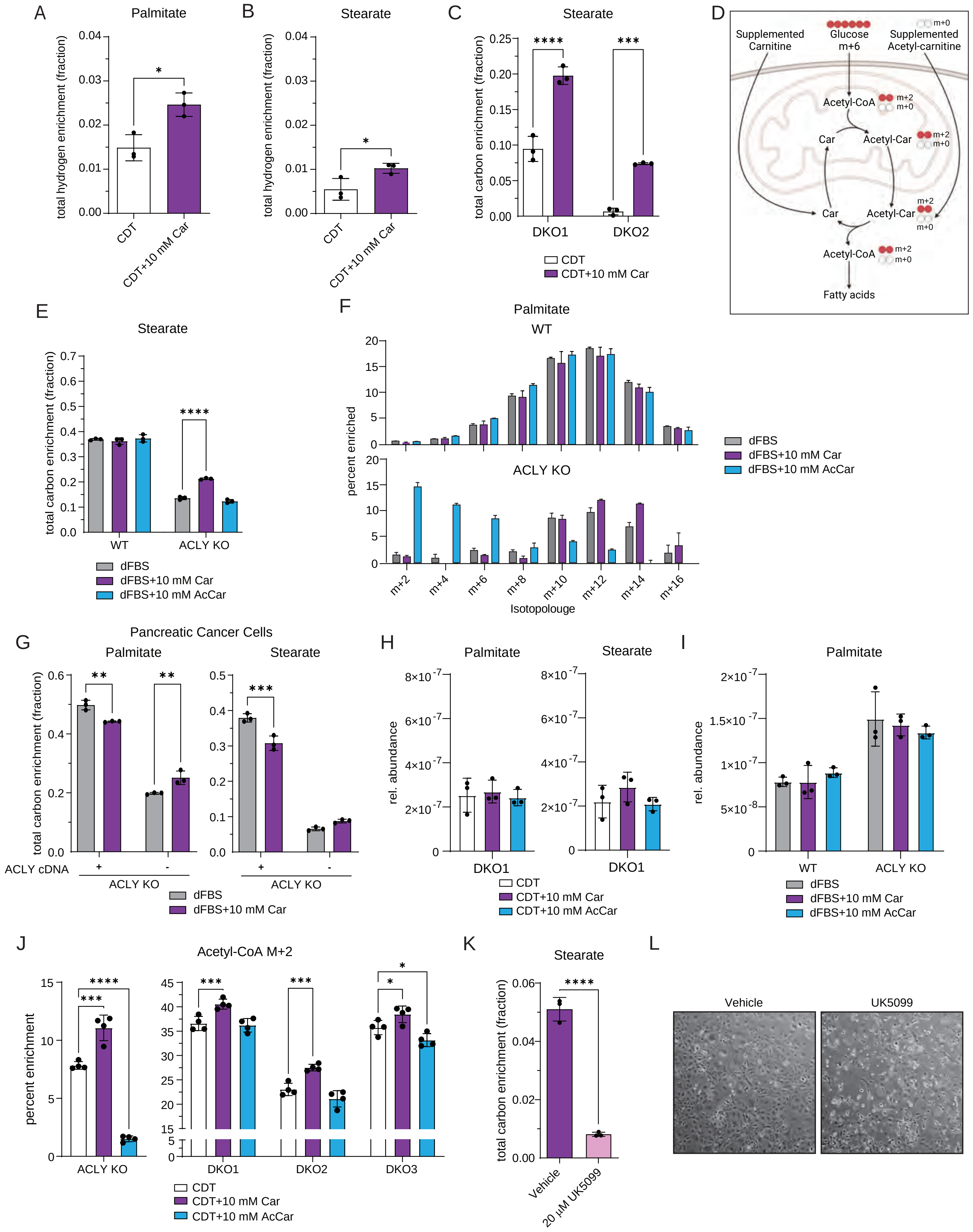

Supplemental Figure 7: Acetylcarnitine shuttling increases glucose derived de novo lipogenesis in the absence of ACLY

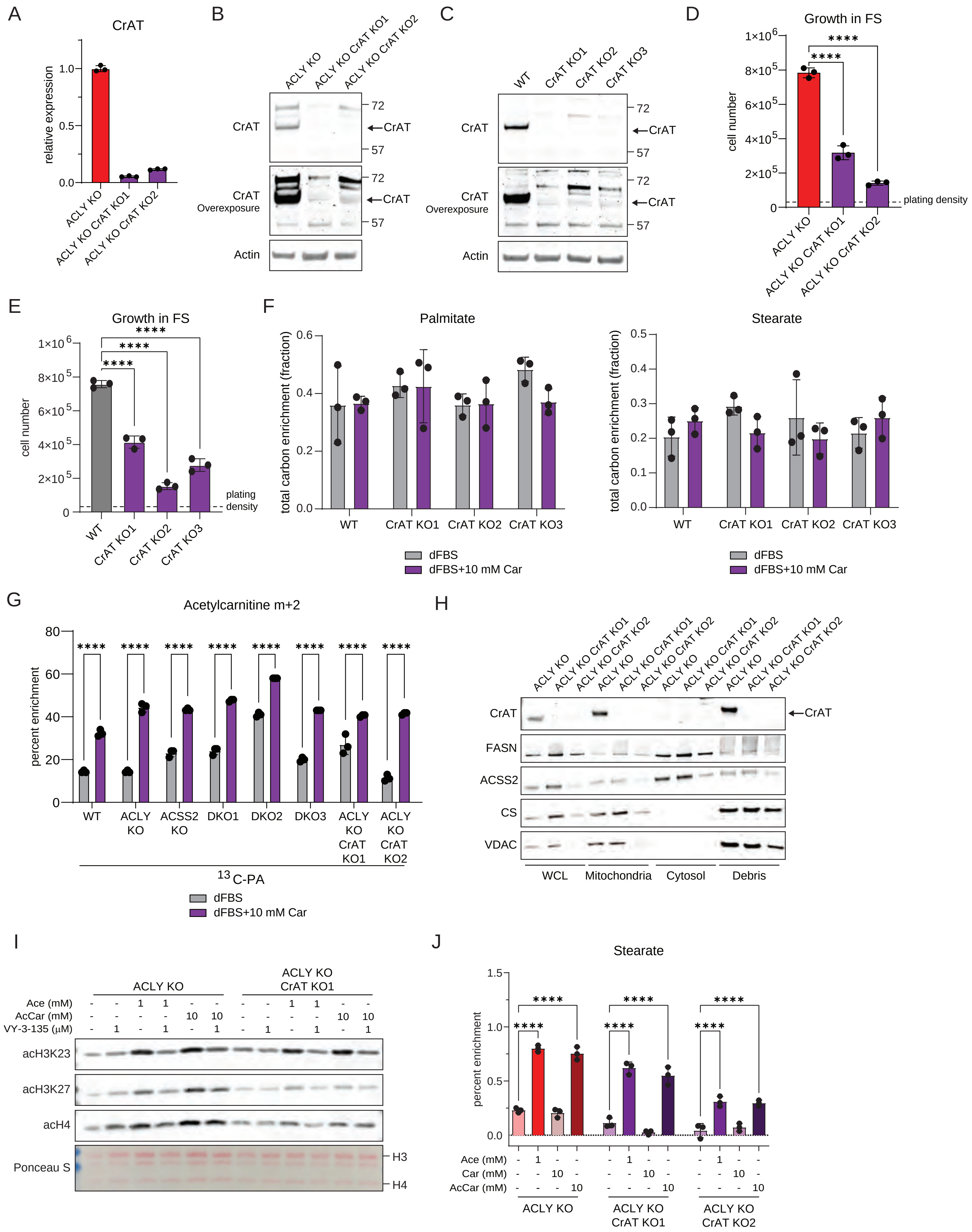

Supplemental Figure 8: CrAT supports acetyl-unit shuttling but is not required for acetylcarnitine to acetyl-CoA conversion

Table S1, Related to Figure 3A

Gene list and cluster ID for WT, ACLY KO, ACSS2 KO, and DKO cells represented on heat map

Symbol	Cluster
Slc6a12	red
Cttnbp2	red
Elfn2	red
Csmd1	red
Taf7l	red
Crhbp	red
Ndst4	red
Trpm5	red
Ly6i	red
Toporsl	red
Npc1l1	red
Ak8	red
Slc47a1	red
Myoz2	red
Pdcd1lg2	red
Megf11	red
Saa3	red
Naip6	red
Sprr2i	red
D830026I12Rik	red
1700019E08Rik	red
Chgb	red
A630095N17Rik	red
Hs3st3a1	red
Adgrl4	red
Pla2r1	red
Dthd1	red
Arsj	red
Acaa1b	red
4930592C13Rik	red
Gm1965	red
Gm10451	red
5430428K19Rik	red
Otogl	red
5430425K12Rik	red
Apol6	red
Gm973	red
A230001M10Rik	red
Gm13031	red
Arhgef6	red
Casq2	red
C330024C12Rik	red
Plek	red

Fam83a	red
Gm1110	red
4930503B20Rik	red
Sox5os3	red
5430427O19Rik	red
Fcgr2b	red
Ceacam10	red
Tmem132e	red
Prl3d2	red
Gm1322	red
Eef1a2	red
Cd177	red
Tigd4	red
Itgam	red
Trpm2	red
Gm8267	red
Gm4861	red
4932414N04Rik	red
4933405E24Rik	red
D930007P13Rik	red
Alpk2	red
Gm28453	red
2810405F15Rik	red
Fgl1	red
Cryba4	red
BC061237	red
9230020A06Rik	red
Pzp	red
Gm4371	red
Alpi	red
Serpinb9f	red
4930509E16Rik	red
Lypd6	red
U90926	red
Kcnn4	red
Sult1d1	red
Atp2c2	red
Gsg1l	red
Coro1a	red
Nps	red
1700018A04Rik	red
Ccdc155	red
Brinp3	red
Cd28	red
5330434G04Rik	red
Hgf	red
Gm2721	red

Nhlrc4	red
Acot12	red
Cngb1	red
Catip	red
Gbx1	red
Olfr875	red
Mill2	red
Olfr225	red
Tnnt3	red
Phf11b	red
Mmp20	red
Gm11529	red
Slc35f1	red
Aim2	red
1700092C10Rik	red
D030045P18Rik	red
Cntn3	red
Umod	orange
Hist1h1t	orange
1600019K03Rik	orange
Kctd14	orange
Hist1h2ad	orange
Cts3	orange
Apof	orange
Mtus2	orange
Stk33	orange
Sprn	orange
Lbx1	orange
Tro	orange
1700113B09Rik	orange
Gm5535	orange
Ugt1a7c	orange
1700012B07Rik	orange
Prelid2	orange
LOC108168459	orange
Nap1l3	orange
C130026I21Rik	orange
Sema5b	orange
44631	orange
Ttc29	orange
Hspb7	orange
Gm12238	orange
Bank1	orange
Lyzl1	orange
Cmtm5	orange
Uba1y	orange
Wnt1	orange

Csf2rb2	orange
9330178D15Rik	orange
Serpina3n	orange
4921511I17Rik	orange
Unc5a	blue
Elavl2	blue
Frzb	blue
Dlx6	blue
Sim1	blue
Shank3	blue
Wipf3	blue
Col23a1	blue
Adamts13	blue
1700007K13Rik	blue
Dlx6os1	blue
B4galnt2	blue
H2-Q1	blue
BB218582	blue
Asxl3	blue
Tubb2a-ps2	light grey
Eif4e3	light grey
Hmga2-ps1	light grey
Zfp354b	light grey
Itgb8	light grey
Gm15050	light grey
Dusp23	light grey
Otulinl	light grey
Cbr3	light grey
Sfrp2	light grey
Pou4f1	light grey
Hddc3	light grey
Ctsf	light grey
Htr1b	light grey
H60b	light grey
Phlda2	light grey
Ccdc30	light grey
Tbx2	light grey
Ms4a4b	light grey
Cdh15	light grey
Crmp1	light grey
Triqk	light grey
Tmem121	light grey
Serpina3j	light grey
1700034H15Rik	light grey
Cfap100	light grey
1700012C14Rik	light grey
Zfp783	light grey

Nfasc	light grey
Rasgef1a	light grey
Tlr6	light grey
Mir3063	light grey
Hmx3	light grey
Gm13648	light grey
Ptgdr2	light grey
Cln3	light grey
Fads6	dark grey
Amph	dark grey
Lhfpl4	dark grey
A030001D20Rik	dark grey
Tdh	dark grey
Ror2	dark grey
Ppp1r14a	dark grey
Gstm4	dark grey
Sp5	dark grey
Galm	dark grey
Hexa	dark grey
Gm8773	dark grey
Dusp9	dark grey
Rhoj	dark grey
Misp	dark grey
Sowahb	dark grey
Cdh26	dark grey
Mansc1	dark grey
Rgs4	dark grey
Drd3	dark grey
